# Mitochondrial Homeostasis in Diabetic Cardiomyopathy: From Dysfunction to Therapeutic Strategies

**DOI:** 10.3390/antiox15030399

**Published:** 2026-03-22

**Authors:** Yafei Huang, Wenyu Zou, Xindi Jiang, Jing Cheng, Jia Zheng

**Affiliations:** 1 Research Center, Peking University First Hospital, Beijing 100034, China; 21434@pkufh.com (Y.H.); p1668@pkufh.com (X.J.); 2 Department of Endocrinology, Peking University First Hospital, Beijing 100034, China; 2211110289@stu.pku.edu.cn; 3 Department of Cardiology, Heart Center, Beijing Chaoyang Hospital, Capital Medical University, Beijing 100020, China; 4 Beijing Key Laboratory of Hypertension, Beijing Chaoyang Hospital, Capital Medical University, Beijing 100020, China

**Keywords:** diabetic cardiomyopathy, mitochondrial homeostasis, pathogenesis

## Abstract

Diabetic cardiomyopathy is a specific form of heart dysfunction that occurs in diabetic patients independent of other cardiomyopathies such as coronary artery disease. It significantly contributes to heart failure and mortality in this population. The pathogenesis of diabetic cardiomyopathy mainly includes oxidative stress, inflammatory response, apoptosis and disrupted mitochondrial homeostasis. Mitochondrial homeostasis, encompassing mitochondrial dynamics, mitochondrial oxidative metabolism and mitophagy, is regulated by a variety of signaling pathways and plays a pivotal role in maintaining the normal function of cardiomyocytes. At present, the exact mechanisms underlying diabetic cardiomyopathy pathogenesis remain unclear, and effective prevention and treatment methods are lacking. This review therefore expounds the pathogenesis of diabetic cardiomyopathy from the perspective of mitochondrial homeostasis, providing new approaches to clinical management.

## 1. Introduction

The prevalence of type 2 diabetes mellitus (T2DM) has been rising steeply and persistently, with the disease now recognized as a global epidemic [1,2,3,4]. In 2021, 529 million individuals had diabetes globally, with projections indicating the number of patients will reach 1.31 billion by 2050 [5,6]. Uncontrolled diabetes leads to a variety of long-term health complications, including cardiovascular disease (CVD), neuropathy, vision problems, and amputation [3,7]. Among these complications, CVD is the leading cause of mortality and morbidity in diabetic patients, accounting for nearly 70% of heart failure cases [8,9,10]. Compared with non-diabetic individuals, diabetic patients face a 2- to 8-fold higher risk of CVD events, primarily due to microvascular and macrovascular atherosclerosis exacerbated by concomitant CVD risk factors including hypertension, dyslipidemia, and the activation of neurohormonal–inflammatory mechanisms [6,8,11].

Diabetic cardiomyopathy (DCM) denotes cardiac dysfunction—initially characterized by diastolic dysfunction with preserved ejection fraction, with potential progression to systolic dysfunction with reduced ejection fraction—observed in patients with diabetes mellitus in the absence of concomitant cardiovascular diseases, including but not limited to coronary artery disease, hypertension, valvular abnormalities, and congenital heart disease [12,13]. Its high prevalence, diagnostic challenges, and poor prognosis profoundly impact patients’ quality of life while being a heavy burden on families and society, driving extensive research into the treatment and prevention of DCM [14,15].

The current understanding of DCM pathogenesis mainly implicates the occurrence of inflammatory responses, changes in calcium signaling, and renin–angiotensin–aldosterone system hyperactivity [16,17,18,19,20]. Recent studies have demonstrated that mitochondrial dysfunction contributes to DCM pathology by disrupting myocardial metabolic and non-metabolic signals, ultimately inducing myocardial dysfunction [21]. This article explores the role of mitochondrial homeostasis in DCM occurrence and progression, reviews current research advances, summarizes its mechanistic involvement in DCM, and establishes a theoretical framework for the clinical treatment of DCM.

## 2. Mitochondrial Homeostasis

Mitochondria, acting as the “powerhouses” of the cell, primarily supply energy for various cellular activities and serve as the central and principal site for cellular metabolism and oxidative respiration. Abnormalities in mitochondrial morphology and function are closely associated with the development of numerous diseases. Mitochondrial homeostasis represents a dynamic regulatory process involving mitochondrial dynamics (fusion and fission), mitochondrial oxidative metabolism, and mitophagy. These coordinated mechanisms maintain both the quantity and structural integrity of “healthy” mitochondria to meet the various energy demands of the cell [22]. Disruption in mitochondrial homeostasis constitutes a key pathological factor in the progression of many diseases. Therefore, maintaining mitochondrial homeostasis is crucial for the normal growth and development of cells and organisms [23].

### 2.1. Mitochondrial Oxidative Metabolism

Mitochondrial oxidative metabolism is a critical process by which cells generate energy in the form of adenosine triphosphate (ATP) through the oxidation of nutrients, which primarily takes place within the mitochondria [24]. Pyruvate, derived from glycolysis, enters the mitochondria and is converted into acetyl-CoA by the pyruvate dehydrogenase complex. Acetyl-CoA then enters the tricarboxylic acid (TCA) cycle, also known as the Krebs cycle, where it is oxidized to produce high-energy electron carriers including nicotinamide adenine dinucleotide (NADH) and flavin adenine dinucleotide (FADH_2_), while releasing carbon dioxide as a waste product [25]. These high-energy electron carriers donate their electrons to the electron transport chain (ETC), which is embedded in the inner mitochondrial membrane. The ETC consists of four protein complexes (Complex I to IV) that transfer electrons sequentially, generating a proton gradient across the inner mitochondrial membrane [26]. As protons flow back into the mitochondrial matrix through ATP synthase (Complex V), the resulting energy drives the synthesis of ATP from adenosine diphosphate (ADP) and inorganic phosphate, thereby coupling the electron transport chain with ATP production.

However, during oxidative metabolism, a small percentage of electrons may leak from the ETC and react with oxygen, forming reactive oxygen species (ROS) such as superoxide radicals. While physiological levels of ROS function as signaling molecules, excessive ROS production induces oxidative stress and damages cellular components, including proteins, lipids, and DNA [26,27].

**Figure 1:**
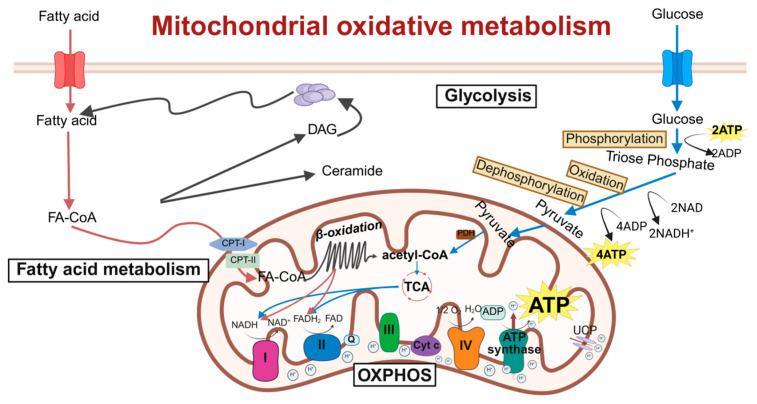
**Schematic of molecular regulatory mechanisms for mitochondrial oxidative metabolism.** This diagram shows key mitochondrial oxidative metabolic processes: the TCA cycle, ETC, and oxidative phosphorylation. Acetyl-CoA is oxidized in the TCA cycle to generate NADH and FADH_2_, which donate electrons to ETC complexes I–IV, pumping protons to form a gradient. Proton re-entry through ATP synthase drives ATP production, converting nutrient energy into usable cellular energy. ETC, electron transport chain; DAG, diacylglycerol; FA-CoA, fatty acyl-coenzyme A; CPT-1, carnitine palmitoyltransferase-1; CPT-2, carnitine palmitoyltransferase-2; NADH, nicotinamide adenine dinucleotide; FAD, flavine adenine dinucleotide, reduced; OXPHOS, oxidative phosphorylation; TCA, tricarboxylic acid cycle; ADP, adenosine diphosphate; ATP, adenosine triphosphate; Cyt c, cytochrome c; PHD, prolyl hydroxylase. Created in BioRender. Zou, W. (2026) https://BioRender.com/8ywx0u1, accessed on 3 March 2026.

Mitochondrial oxidative metabolism is precisely regulated by multiple factors, such as substrate availability, hormonal signals, and cellular energy demands. For instance, during fasting or exercise, the expression of oxidative metabolism-related genes increases to meet elevated energy requirements. The dysregulation of mitochondrial oxidative metabolism is linked to numerous diseases, including metabolic disorders, neurodegenerative diseases, cardiovascular diseases, and aging-related conditions [28,29]. Therefore, understanding the mechanisms and regulation of mitochondrial oxidative metabolism is essential for developing therapeutic interventions to restore cellular energy balance and mitigate oxidative stress (Figure 1).

### 2.2. Mitochondrial Dynamics

Mitochondria are highly dynamic organelles that undergo continuous cycles of fusion and fission to modulate their morphology, size, and location. This physiological process is known as mitochondrial dynamics [30]. The relative balance between mitochondrial fission and fusion is crucial for maintaining mitochondrial quality and function, which is of great significance to normal cellular activities [31,32].

Mitochondrial fission is primarily regulated by dynamin-related protein 1 (DRP1) [33]. DRP1, a member of the dynamin superfamily, is typically localized in the cytoplasm and comprises a GTP hydrolysis (GTPase) domain, a middle domain, and a GTPase effector domain. Upon stimulation by fission factors, DRP1 is recruited to the mitochondria, where it oligomerizes to form ring-like structures. These structures, utilizing the GTPase activity of DRP1, induce the scission of the mitochondrial outer and inner membranes, resulting in mitochondrial fission [34]. The receptors for DRP1 include mitochondrial fission factor (MFF), fission1 (FIS1), mitochondrial dynamics proteins of 49 kDa (MiD49), and mitochondrial dynamics proteins of 51 kDa (MiD51), which mediate both the recruitment of DRP1 and the process of mitochondrial fission [35]. Among these, MFF is a predominant receptor for DRP1 in mammalian cells. The overexpression of MFF promotes mitochondrial fission, whereas its knockdown leads to mitochondrial elongation [36]. Inorganic pyrophosphatase phosphatase 2A (PPA2), a protein localized to the mitochondrial matrix, activates the downstream fission signaling pathway by directly interacting with the mitochondrial inner-membrane protein mitochondrial fission process 1 (MTFP1). Specifically, the overexpression of PPA2 significantly promotes the phosphorylation of DRP1 at serine 616 and enhances the recruitment of phosphorylated DRP1 to mitochondria, thereby initiating the fission process. When MTFP1 is knocked down, even the overexpression of PPA2 cannot induce the activation of DRP1 and subsequent fission, confirming the necessity of MTFP1 in this pathway [37]. It has also been reported that MTFP1 itself is not a pro-fission factor; instead, it functions by inhibiting fusion, thereby “isolating” damaged or dysfunctional inner-mitochondrial-membrane (IMM) subdomains from the healthy mitochondrial network. Subsequently, these isolated subdomains are separated into small MTFP1-enriched mitochondria (SMEM) via peripheral fission and are ultimately degraded through the autophagy pathway [38].

**Figure 2:**
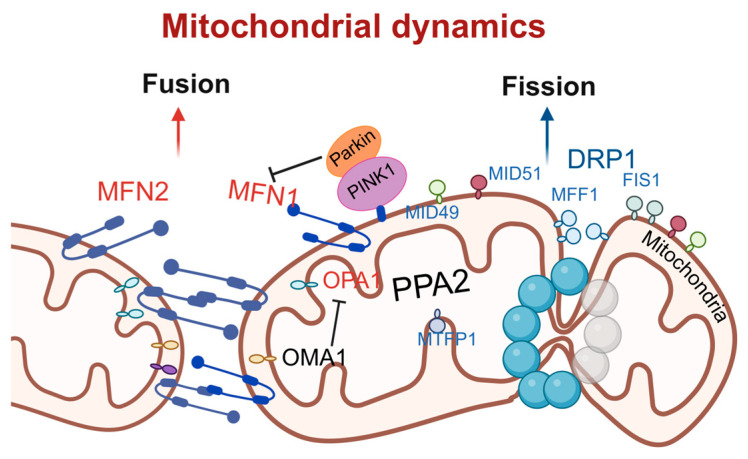
**Schematic of molecular regulatory mechanisms for mitochondrial fusion and fission.** Mitochondrial fusion is mediated by MFN1/2 and OPA1 and negatively regulated by PINK1/Parkin; fission is driven by DRP1 and its receptors FIS1, MFF, and others. The dynamic equilibrium between fusion and fission maintains mitochondrial function and cellular homeostasis. DRP1, dynamin-related protein 1; MFF, mitochondrial fission factor; FIS1, fission1; MID49, mitochondrial dynamics proteins of 49 kDa; MID51, mitochondrial dynamics proteins of 51 kDa; PINK1, PTEN-induced kinase 1; Parkin, E3 ubiquitin–protein ligase parkin; PPA2, phosphatase 2A; MTFP1, mitochondrial fission process 1; OMA1, overlapping with the M-AAA protease 1 homolog; OPA1, optic atrophy 1; MFN1, mitofusin 1; MFN2, mitofusin 2. Created in BioRender. Zou, W. (2026) https://BioRender.com/9ojsyrq, accessed on 3 March 2026.

Mitochondrial fusion is a multistep process that includes mitochondrial tethering, outer-membrane fusion, and inner-membrane fusion [39]. The fusion process is primarily regulated by mitochondrial fusion proteins, including mitofusin 1 (MFN1), mitofusin 2 (MFN2), and optic atrophy 1 protein (OPA1) [40]. MFN1 and MFN2 mainly mediate the fusion of the mitochondrial outer membrane by forming homodimers or heterodimers through cis-dimerization, thereby facilitating the tethering and fusion of the outer membranes of adjacent mitochondria [41,42]. OPA1 is primarily involved in the fusion of the mitochondrial inner membrane. Its gene deletion leads to mitochondrial fragmentation, while overexpression results in mitochondrial elongation [43]. OPA1 induces inner-membrane fusion in a manner dependent on MFN1 but not MFN2, indicating potential communication and interaction between the outer and inner mitochondrial membranes [43]. However, the exact mechanism by which OPA1 induces inner-membrane fusion remain incompletely understood and requires further investigation. Recent studies have revealed a synergistic role of two classic mitochondrial stress response systems, the PTEN-induced kinase 1 (PINK1)/E3 ubiquitin–protein ligase parkin (Parkin) pathway and the metalloprotease overlapping with the M-AAA protease 1 homolog (OMA1), in cooperatively inhibiting excessive mitochondrial fusion under physiological conditions, thereby maintaining mitochondrial structural and genomic integrity. The PINK1/Parkin pathway primarily targets the outer-membrane fusion protein MFN1, promoting its ubiquitination and degradation to inhibit outer-membrane fusion. OMA1 primarily targets the inner-membrane fusion protein OPA1, inactivating it through proteolytic cleavage to inhibit inner-membrane fusion. The individual deletion of either Parkin or OMA1 does not affect mitochondrial integrity, but the combined deletion of both leads to excessive mitochondrial fusion, forming megamitochondria, which are particularly prominent in the brainstem and heart [44] (Figure 2).

### 2.3. Mitophagy

Mitophagy is a selective process by which cells remove damaged or dysfunctional mitochondria through autophagy, playing a crucial role in maintaining cellular homeostasis and mitochondrial quality control. The main pathways of mitophagy include ubiquitin-mediated mitophagy, receptor-mediated mitophagy and mitochondrial-derived vesicles (MDVs) pathways.

Receptor-mediated mitophagy is initiated by the direct interaction of the outer-mitochondrial-membrane (OMM) proteins with microtubule-associated protein 1 light chain 3 (LC3)/gamma–aminobutyric acid receptor-associated protein (GABARAP) proteins on the autophagosome membrane through the LC3 interaction region (LIR) motif [45]. PINK1 and Parkin, two proteins associated with Parkinson’s disease, have recently been identified as regulators of mitophagy [46]. PINK1 functions as an upstream regulator of Parkin, collaborating with it to promote the autophagic degradation of damaged mitochondria via the polyubiquitination of mitochondrial surface proteins. Following mitochondrial depolarization or damage, PINK1 accumulates extensively on OMM, triggering the translocation of Parkin from the cytosol to the OMM [47]. Parkin, leveraging its E3 ligase activity, conjugates ubiquitin to substrate proteins, forming polyubiquitin chains [46,48]. Subsequently, adaptor proteins such as p62/sequestosome-1 are recruited to the mitochondria through their ubiquitin-binding domains and interact with LC3, facilitating the aggregation of ubiquitinated proteins into autophagosomes for degradation [48]. Moreover, the PINK1/Parkin pathway is involved in an unconventional autophagic process, where specific proteins are transported to lysosomes for degradation via MDVs [49].

Receptor-mediated mitophagy is initiated by the direct interaction of mitochondrial OMM proteins with LC3/GABARAP proteins on the autophagosome membrane through the LIR motif [45]. NIP3-like protein X (NIX) and BCL2/adenovirus E1B 19 kDa interacting protein 3 (BNIP3), as members of the B cell lymphoma 2 (Bcl-2) family, are OMM-localized proteins that mediate mitophagy through their N-terminal WXXL-like sequences that bind to LC3. NIX is involved in mitochondrial clearance during reticulocyte maturation, whereas BNIP3 participates in hypoxia-induced mitophagy and plays a key role in the induction and maintenance of pluripotency [50,51]. Under hypoxic conditions, hypoxia-inducible factor-1 upregulates the expression of NIX and BNIP3, disrupting the interaction between Bcl-2 and Beclin-1 and activating mitophagy [52]. FUN14 domain containing 1 (FUNDC1), another receptor protein for hypoxia-induced mitophagy, also interacts with LC3 via its WXXL-like sequence, with its phosphorylation status precisely controlling mitophagy initiation [53]. When mitophagy is induced, FUNDC1 dissociates from OPA1 and binds to DRP1, subsequently leading to mitochondrial fission and mitophagy. It has been reported that FUNDC1 plays a role in cardiac progenitor cell differentiation by remodeling the mitochondrial network and exerts cardioprotective effects during ischemia–reperfusion injury [54]. Collectively, these proteins orchestrate the removal of mitochondria damaged by hypoxia, thereby maintaining oxygen homeostasis and mitigating ROS accumulation [55].

**Figure 3:**
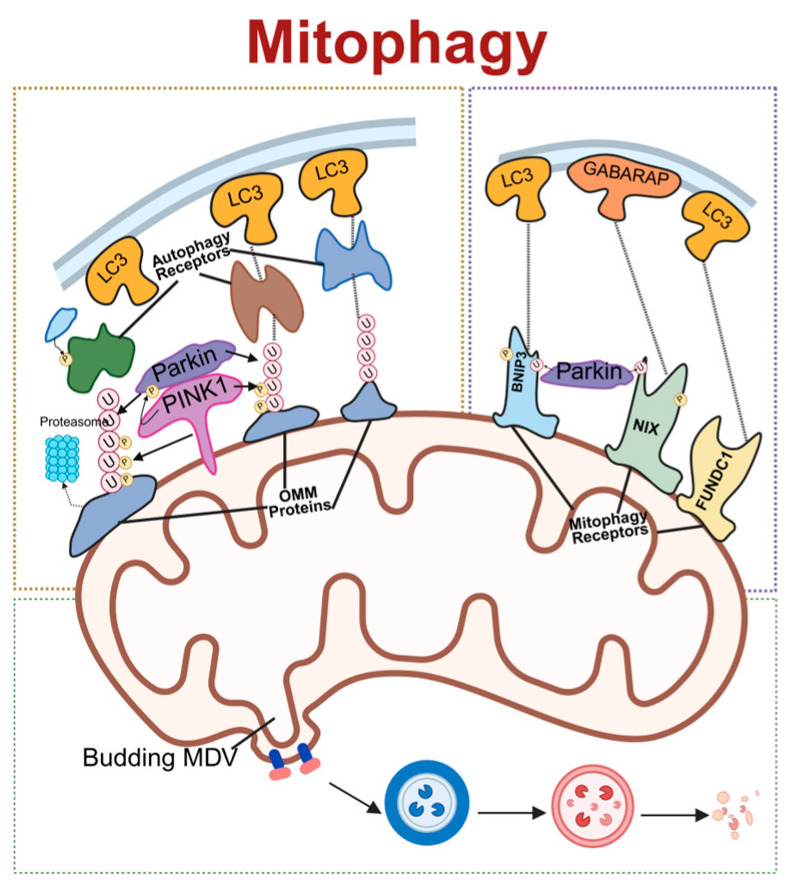
**Schematic of molecular regulatory mechanisms for mitophagy.** The three primary pathways of mitochondrial autophagy: On the left is the PINK1/Parkin-dependent pathway, where PINK1 recruits Parkin to ubiquitinate outer-mitochondrial-membrane proteins, subsequently recruiting autophagy receptors that bind LC3. On the right is the receptor-mediated pathway, where receptors such as Nix, BNIP3, and FUNDC1 directly bind to LC3/GABARAP. Below is the mitochondrial-derived vesicle (MDV) pathway. Together, these three pathways mediate the selective autophagic degradation of mitochondria. LC3, microtubule-associated protein 1 light chain 3; PINK1, PTEN-induced kinase 1; Parkin, E3 ubiquitin–protein ligase parkin; OMM, outer mitochondrial membrane; MDV, mitochondrial-derived vesicle; BNIP3, BCL2/adenovirus E1B 19 kDa interacting protein 3; NIX, NIP3-like protein X; FUNDC1, FUN14 domain containing 1; GABARAP, gamma–aminobutyric acid receptor-associated protein. Created in BioRender. Zou, W. (2026) https://BioRender.com/epmyj4i, accessed on 3 March 2026.

MDV formation is a key pathway in mitochondrial quality control. MDV biogenesis complements mitophagy and serves to recycle damaged mitochondria [56]. When the primary mitophagy mechanism is impaired due to aging or other conditions, mitochondrial Rho GTPase1/2 proteins located on the mitochondrial outer membrane sense signals and recruit the dynamin-related protein DRP1, driving local constriction and budding of the mitochondrial membrane to initiate MDV formation [57]. Meanwhile, a decrease in damaged mitochondrial membrane potential can activate the PINK1/Parkin pathway: PINK1 stabilizes on the outer mitochondrial membrane, and recruits and activates the E3 ubiquitin ligase Parkin, which then ubiquitinates outer membrane proteins to tag damaged components [58]. The adaptor protein Tollip recognizes these ubiquitination signals, assists in recruiting autophagy-related proteins, and sorts specific cargo (such as oxidatively damaged proteins, lipids, and mitochondrial DNA) into the forming MDVs [54,59]. Finally, mature MDVs fuse with late endosomes/lysosomes via sensitive factor attachment protein receptors proteins such as syntaxin-17 on their membrane, delivering their contents to the lysosome for degradation [60]. This process is independent of canonical macroautophagy/mitophagy and represents an important supplementary mechanism for cells to clear oxidatively damaged mitochondrial components and maintain mitochondrial network integrity (Figure 3).

## 3. Mitochondrial Homeostasis and DCM

**Figure 4:**
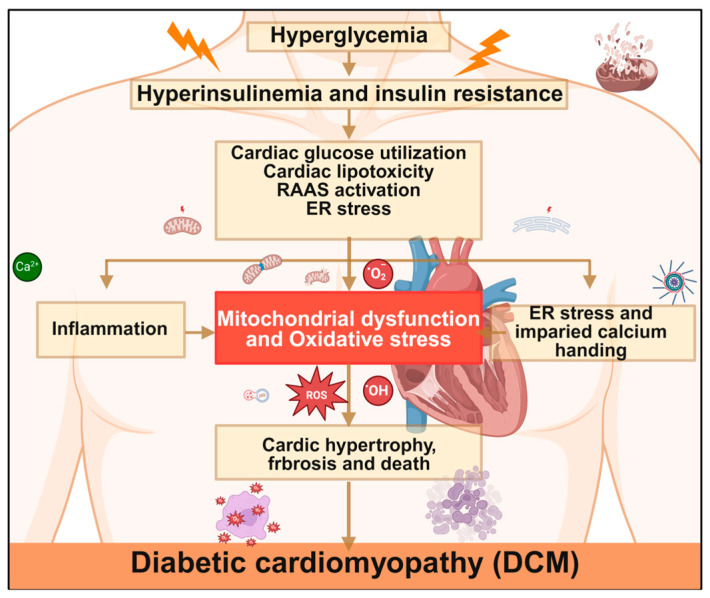
**Mechanisms of diabetic cardiomyopathy.** Hyperglycemia triggers cardiac metabolic disorders through hyperinsulinemia and insulin resistance, leading to mitochondrial dysfunction and oxidative stress. This subsequently induces inflammation, endoplasmic reticulum stress, and impaired calcium handling, ultimately promoting the development of DCM. RAAS, renin–angiotensin–aldosterone system; ER, endoplasmic reticulum; ROS, reactive oxygen species. Created in BioRender. Zou, W. (2026) https://BioRender.com/cmufxug, accessed on 3 March 2026.

In the pathogenesis and progression of DCM, mitochondrial dysfunction constitutes a core pathological mechanism involving multiple levels such as impaired oxidative metabolism, kinetic abnormalities, and disrupted autophagy (Figure 4 and Table 1). The following provides a systematic elaboration on key molecular events.

### 3.1. Mitochondrial Oxidative Metabolism and DCM

Mitochondrial oxidative metabolism refers to the central mitochondrial pathway where sugars, fats and amino acids undergo oxidative phosphorylation through the tricarboxylic acid cycle, ultimately generating ATP through redox reaction to meet energy demands. Ji et al. [62] demonstrated that mitochondrial calcium uptake 1 (MICU1) serves as a key regulator of mitochondrial Ca^2+^ uptake and critically modulates mitochondrial oxidative phosphorylation and redox homeostasis by elevating mitochondrial Ca^2+^. This mechanism activates the antioxidant system, thereby inhibiting the apoptosis of cardiomyocytes induced by high sugar and high lipids, and exerts a protective effect on myocardium.

As highly energy-dependent cells, cardiomyocytes require continuous oxidative metabolism to generate ATP necessary for maintaining cardiac output and contractile function [77]. Emerging evidence suggests that Ca^2+^ homeostasis is closely related to mitochondrial energy metabolism, functioning as an essential second messenger to maintain proper cardiac function [78,79]. Notably, abnormal Ca^2+^ signaling can be observed in type 2 diabetic DCM, with Ca^2+^ mishandling being the main reason for an interference in energy production. Studies reveal that significantly elevated pyruvate dehydrogenase phosphorylation in diabetic cardiomyocytes alters the mitochondrial energy metabolism and counteracts mitochondria-associated membrane Ca^2+^ signaling. Its correction could restore cardiac function and prevent DCM progression.

Mitochondrial oxidative metabolism dysfunction is accompanied by a large amount of ROS production, mitochondrial peroxidative damage, and myocardial injury due to energy insufficiency, constituting a key pathological mechanism in DCM. Li et al. [80] reported that T2DM exacerbates transient receptor potential vanilloid 1 (TRPV1) blockade and ROS overload, leading to cardiac microvascular injury. Notably, inhibiting TRPV1/Ca^2+^-mediated oxidative/nitrifying stress response effectively protected cardiac microvessels from diabetic damage. Excess ROS can, in turn, activate the p53/synthesis of cytochrome c oxidase 2 (SCO2) signaling, increasing mitochondrial oxygen consumption, and perpetuating ROS production and lipid accumulation, thereby driving diabetic myocardial injury [81].

Berthiaume et al. [82] identified that diabetic cardiomyocytes display “metabolic rigidity” with pathologically enhanced fatty acid uptake and mitochondrial oxidation, despite the fact that the healthy adult heart already relies primarily on fatty acids for energy (following the normal developmental switch from glucose that occurs after birth). This rigidity is characterized by impaired mitochondrial electron transport chain function, insulin resistance-mediated suppression of glucose transporters, and, consequently, markedly reduced glucose utilization. The transformation of energy metabolism changes the transcriptional and redox status of NAD and metabolite signaling of key enzymes, resulting in a decrease in glycolysis and mitochondrial oxidative metabolism that can cause cardiac energy deficiency and cardiac insufficiency [83]. Furthermore, A-kinase-anchored protein 121 increases cardiomyocyte apoptosis by enhancing ROS production, and deficiency of this protein impairs mitochondrial respiration, reduces ATP production, and induces mitochondrial oxidative metabolism dysfunction, along with cardiomyocyte apoptosis [84]. Studies have demonstrated that reduced NADPH oxidase 5, a Ca^2+^-sensitive, pro-contractile NADPH oxidase isoform, can be mediated to participate in oxidative stress regulation, resulting in an increase in ROS levels. Activation of the mitogen-activated protein kinase pathway leads to cardiac hypertrophy and systolic dysfunction [85]. Collectively, Ca^2+^ affects myocardial oxygen consumption by regulating mitochondrial energy metabolism in cardiomyocytes, induces oxidative stress, and changes cardiac structure and function. In summary, mitochondrial oxidative metabolic dysfunction plays a pivotal role in DCM. The dysregulation of mitochondrial oxidative metabolism leads to excessive production of ROS and peroxidative damage to mitochondria, thereby causing myocardial energy deficiency and myocardial injury. Moreover, the disruption of Ca^2+^ homeostasis further interferes with mitochondrial energy metabolism, exacerbating myocardial dysfunction and ultimately leading to alterations in cardiac structure and function.

Importantly, the aged myocardium provides a vulnerable substrate for diabetic metabolic insults [86]. Cardiac aging is characterized by mitochondrial metabolic inflexibility, including a diminished capacity for fatty acid oxidation and compromised glucose utilization, which predisposes the heart to energetic deficiency [87]. Concurrently, aged mitochondria exhibit increased electron leak and a decline in antioxidant defense systems, creating a state of basal oxidative stress [88]. When diabetic conditions—such as glucotoxicity, lipotoxicity, and insulin resistance—are superimposed on this aged milieu, they act synergistically to exacerbate mitochondrial ROS production and overwhelm cellular antioxidant capacity [89]. This age-aggravated metabolic dysregulation and oxidative stress not only accelerate ATP depletion but also inflict more severe damage on proteins, lipids, and mtDNA, thereby propelling the progression of diastolic dysfunction and myocardial fibrosis characteristic of advanced DCM [90,91].

### 3.2. Mitochondrial Dynamics and DCM

Mitochondrial dynamics refers to the process that shape the mitochondrial network, regulate mitochondrial function, and maintain quality control. This dynamic process integrates mitochondrial fusion and fission with diverse cellular functions while responding to cellular pathophysiological changes. Strategically balancing these two processes demonstrates therapeutic potential for DCM improvement [92].

Previous studies have shown that increased fission and decreased fusion can aggravate mitochondrial fragmentation, in which fission-mediated mitochondrial fragmentation directly contributes to high-glucose-induced ROS overproduction, whereas fusion counteracts this damage by facilitating complementation between damaged mitochondria [93]. Dai and Jiang [94] found that mitochondrial fusion is controlled by three guanosine triphosphate (GTP) enzymes, and fission is mainly regulated by GTPase kinetics-related protein 1. The decrease in GTPase activity will inhibit mitochondrial dynamics and cellular metabolism, predisposing to metabolic diseases such as DCM.

In diabetic conditions, increased mitochondrial fission can lead to myocardial damage and systolic dysfunction. Hu et al. [69] found that diabetic myocardium exhibits excessive mitochondrial fission, which can be suppressed by mitochondrial fusion, thereby promoting the recovery of mitochondrial membrane potential and oxidative stress, and arresting DCM progression. Similarly, Ding et al. [95] found that diabetic myocardial systolic dysfunction is associated with increased mitochondrial fission, and that melatonin regulates the protein 1/peroxisome proliferator-activated receptor γ coactivator 1-alpha (PGC-1α) pathway by silencing, reduces the expression of GTPase kinetics-related protein 1, prevents mitochondrial fission, and alleviates diabetes-induced cardiac dysfunction. Li et al. [96] reported that Ophiopogonin D alleviates mitochondrial damage and dysfunction by inhibiting mitochondrial fission in cardiomyocytes, improving cell survival, and preventing and treating diabetic myocardial injury. In summary, the targeted modulation of mitochondrial dynamics reduces pathological ROS release, alleviates myocardial oxidative stress, inflammatory response and fibrosis, and ultimately improves myocardial injury and dysfunction in diabetes.

The regulation of mitochondrial dynamics is further compromised by the aging process itself [88]. In the senescent heart, a baseline shift towards excessive fission (elevated DRP1 activity) and impaired fusion (reduced MFN2 expression) is commonly observed, leading to a progressive fragmentation of the mitochondrial network even in the absence of diabetes [97,98]. The diabetic milieu, with its associated oxidative stress and activation of kinases like cyclin-dependent kinases (CDK1)/Cyclin B, further amplifies DRP1-mediated fission and impairs OPA1-mediated inner membrane fusion [89,99]. Thus, aging and diabetes converge on the same molecular machinery, creating a feed-forward cycle that drives the mitochondrial network toward a highly fragmented, dysfunctional state.

### 3.3. Mitophagy and DCM

Mitophagy refers to the selective removal of damaged and dysfunctional mitochondria, which helps to improve energy metabolism while regulating the quantity of mitochondria so as to maintain the basic quality of cardiac mitochondria. Mitophagy is affected by misfolded protein accumulation, mitochondrial dysfunction, and the deletion of gene fragments [100]. There is a large body of evidence that mitophagy can improve cardiac function in diabetic patients, and the loss of mitophagy is strongly correlated to DCM development [101].

Mu et al. [76] found that the upregulation of bromodomain-containing protein 4 in the heart of diabetic mice suppresses PINK1 expression, inhibiting PINK1/Parkin-mediated mitophagy and causing damaged mitochondria accumulation, resulting in impaired cardiac structure and function that aggravate cardiomyopathy. It also reduces the therapeutic effect of bromodomain-containing protein 4 inhibitors, which restores mitophagy by inhibiting the upregulation of bromodomain-containing protein 4 and repairs cardiac structure and function in diabetic hearts. Metformin alleviates low-grade inflammatory responses through blocking mitochondrial complex 1, inhibiting inflammasome activation, increasing autophagy and improving mitochondrial bioenergy metabolism. It also inhibits the mammalian target protein pathway of rapamycin, alleviates pyroptosis of DCM, and exerts cardioprotective effects [102,103].

Tong et al. [75] revealed that impaired mitophagy induces mitochondrial dysfunction and lipid deposition to aggravate DCM, while mitophagy activation prevents high-fat-diet-induced DCM. The inhibition of mitophagy leads to the excessive accumulation of malformed and inefficient mitochondria, disrupting mitochondrial quality control and redox balance, and ultimately compromising myocardial function [104]. Tahrir et al. [105] further established that mitophagy isolates unhealthy mitochondria and maintains cardiac homeostasis by selectively eliminating damaged or dysfunctional mitochondrial proteins. Meanwhile, Chen et al. [106] identified Ca^2+^ as a key modulator of DCM pathogenesis through directly or indirectly involved in the regulatory mechanisms. Collectively, mitophagy is involved in various physiological and pathological processes such as cardiomyocyte metabolic activity, cell differentiation, and apoptosis, playing an important role in the regulation of cardiovascular diseases [107].

The efficiency of mitophagy, a critical line of defense, is susceptible to the dual assault of both aging and diabetes [108,109]. Aging is associated with a global decline in autophagic flux, attributed to reduced lysosomal function and impaired autophagosome formation. This results in an accumulation of dysfunctional, ROS-producing mitochondria at baseline [86]. Diabetic stress, through mechanisms such as the oxidative modification of Parkin or inhibition of the PINK1/Parkin pathway, directly suppresses the activation of targeted mitophagy. The synergistically impaired mitophagy allows for the unchecked proliferation of damaged mitochondria, leading to overwhelming oxidative stress, activation of the NOD-like receptor family pyrin domain containing 3 (NLRP3) inflammasome, and the release of pro-apoptotic factors [108]. This axis represents a pivotal convergence point where aging exacerbates diabetic injury, and restoring mitophagy may hold particular therapeutic promise for the elderly DCM population [109].

In summary, mitochondrial damage exerts regulatory effects in multiple links of DCM. For example, it leads to abnormal mitochondrial energy metabolism, increased ROS production, mitochondrial fission and fusion disorders, cardiolipin changes, and calcium disorders. These collectively contribute to cardiac dysfunction, coronary microvascular disease, and cardiac structural changes, thereby accelerating the progression of DCM. Conversely, DCM perpetuate mitochondrial functional damage, creating a vicious cycle where these processes mutually reinforce disease progression.

## 4. Pharmacological Treatment

**Figure 5:**
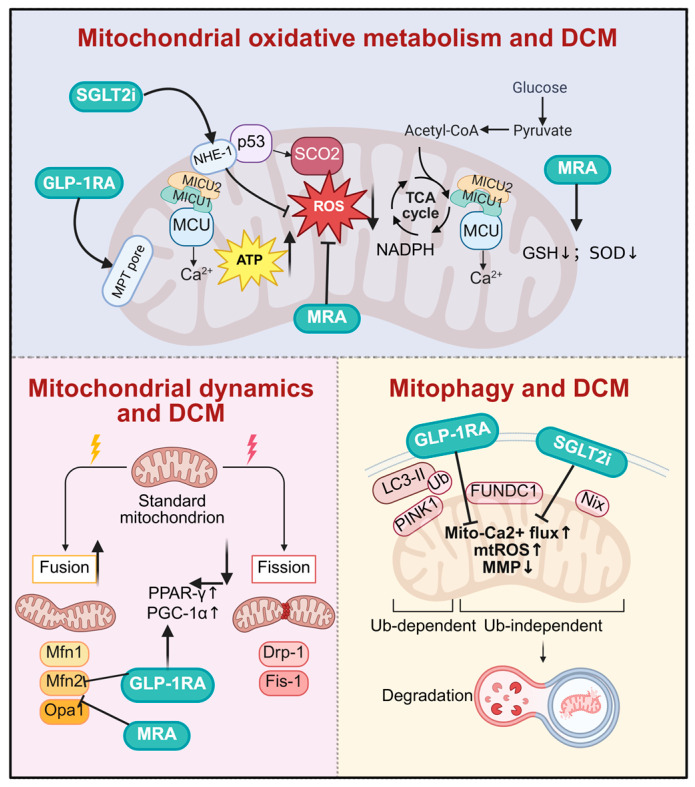
**Drugs target mitochondrial homeostasis for diabetic cardiomyopathy.** SGLT2i, GLP-1RA, and MRA improve DCM by multidimensionally regulating mitochondrial function. At the oxidative metabolism level, SGLT2i and GLP-1RA stabilize mitochondrial Ca^2+^ homeostasis, reduce oxidative stress, and promote ATP production. In mitochondrial dynamics, all three classes synergistically promote mitochondrial fusion and inhibit excessive fission. Regarding mitochondrial autophagy, they accelerate the clearance of damaged mitochondria by activating both PINK1/Parkin-dependent and FUNDC1-mediated independent pathways, thereby exerting comprehensive cardioprotective effects. DCM, diabetic cardiomyopathy; SGLT2i, sodium–glucose cotransporter 2 inhibitors; GLP-1RA, glucagon-like peptide-1 receptor agonist; MRA, mineralocorticoid receptor antagonists; NHE-1, sodium/hydrogen exchanger; MICU1, mitochondrial calcium uptake 1; MICU2, mitochondrial calcium uptake 2; MCU, mitochondrial calcium uniporter; p53, tumor protein p53; SCO2, synthesis of cytochrome C oxidase 2; MPT pore, mitochondrial permeability transition pore; GSH, glutathione; SOD, superoxide dismutase; NADPH, nicotinamide adenine dinucleotide phosphate; PPAR-γ, peroxisome proliferator-activated receptor γ; PGC-1α, peroxisome proliferator-activated receptor gamma coactivator 1-alpha; mtROS, mitochondrial reactive oxygen species; MMP, mitochondrial membrane potential. Created in BioRender. Zou, W. (2026) https://BioRender.com/tpghv6y, accessed on 3 March 2026.

DCM is a complication of diabetes mellitus that develops independently of coronary artery disease and hypertension. It is characterized by alterations in myocardial structure and function, progressing inevitably to heart failure. The underlying pathogenesis of DCM is complex, with no specific therapies currently available. Pharmacological treatment focuses on glycemic control, improvement in cardiac function, prevention of complications, and retardation of disease progression (Figure 5 and Table 2).

### 4.1. Sodium–Glucose Co-Transporter 2 (SGLT2) Inhibitors

SGLT2 inhibitors are a new class of glucose-lowering drugs primarily acting by inhibiting renal glucose reabsorption, thereby promoting urinary glucose excretion and lowering blood sugar [122,123,124,125,126]. Empagliflozin exerts cardioprotective effects in DCM by enhancing mitochondrial ketone body metabolism (upregulating 3-hydroxybutyrate dehydrogenase 1(BDH1)/3-Oxoacid CoA-Transferase 1 (OXCT1)) and attenuating oxidative stress via nuclear factor erythroid 2-related factor 2 (NRF2) activation, thereby reducing ROS production and apoptosis in db/db mouse hearts [110,127,128,129]. Additionally, dapagliflozin exerts anti-cardiac fibrosis effects independent of its hypoglycemic action by inhibiting the endothelial-to-mesenchymal transition (EndMT) to block fibroblast recruitment and directly suppressing cardiac fibroblast proliferation, activation, and collagen synthesis. Its cardioprotective efficacy is comparable to that of metformin [111,130,131]. Canagliflozin improves DCM by activating PINK1/Parkin-dependent mitochondrial autophagy. This study systematically elucidates its protective mechanism from the cellular to animal levels and validates key targets using PINK1 knockdown, providing new insights into the cardioprotective effects of SGLT2 inhibitors. However, its dose-dependent nature and long-term efficacy require further clinical validation [112,132].

SGLT2 inhibitors exert direct cardioprotective effects through a variety of mechanisms, including improved mitochondrial function, anti-inflammatory actions, anti-fibrotic effects, and the mitigation of oxidative stress [133,134,135]. Among these, the impact of SGLT2 inhibitors on mitochondrial function is particularly noteworthy [136]. Pioglitazone exerts its cardioprotective effects through the peroxisome proliferator-activated receptor γ (PPAR-γ)/PGC-1α signaling axis. The knockdown of PGC-1α completely abolished its ability to upregulate Mn Superoxide dismutase (Mn-SOD) and stabilize mitochondrial membrane potential (MMP), confirming that PGC-1α is a key and essential molecular target mediating pioglitazone’s improvement in mitochondrial oxidative stress and prevention of diabetic atrial remodeling [113,137]. Chen et al. (2024) [138] demonstrated that empagliflozin (Empa) ameliorates cardiac dysfunction through inhibiting sodium–hydrogen exchanger 1 (NHE-1), thereby reducing intracellular sodium and calcium overload, decreasing mitochondrial ROS production, and preserving nitric oxide (NO) bioavailability. These effects ultimately improve cardiac contractile function and vascular endothelial metabolic homeostasis, protecting cardiomyocytes from oxidative stress and energy metabolic imbalance, with the underlying mechanism involving the NHE-1/protein kinase B (AKT)/endothelial nitric oxide synthase (eNOS) signaling pathway.

In recent years, SGLT2 inhibitors have shown potential in the treatment of DCM. The results of the EMPA-REG OUTCOME (Empagliflozin Cardiovascular Outcome Event Trial in Type 2 Diabetes Mellitus Patients) trial showed that patients with T2DM at high risk for cardiovascular events who received empagliflozin had a lower rate of the primary composite cardiovascular outcome and a lower rate of death from any cause when the study drug was added to standard care [139,140]. In EMPA-HEART CardioLink-6 clinical trial, among people with T2DM and coronary artery disease, SGLT2 inhibition with empagliflozin was associated with a significant reduction in left ventricular mass indexed to body surface area after 6 months [141]. Clinical trials have unequivocally demonstrated benefits in heart failure across the ejection fraction spectrum. Empagliflozin reduced the composite risk of cardiovascular death or hospitalization for heart failure (HF) in patients with heart failure and preserved ejection fraction (HFpEF) in the EMPEROR-Preserved trial (Empagliflozin Outcome Trial in Patients with Chronic Heart Failure with Preserved Ejection Fraction) [142]. Similarly, dapagliflozin improved outcomes in patients with HFpEF and mildly reduced ejection fraction in the DELIVER (Dapagliflozin Evaluation to Improve the Lives of Patients With Preserved Ejection Fraction Heart Failure) trial [143], and reduced mortality and HHF in patients with heart failure and reduced ejection fraction (HFrEF) in the DAPA-HF (Dapagliflozin and Prevention of Adverse Outcomes in Heart Failure) trial [144]. The consistency of benefit irrespective of diabetes status underscores that SGLT2 inhibitor effects extend beyond glycemia control.

Looking ahead, SGLT2 inhibitors are in a position to play a significant role in the treatment of DCM through their ability to enhance mitochondrial function, mitigate oxidative stress and regulate inflammatory responses. These multifaceted effects establish them as a therapeutic strategy that protects cardiac function, improves vascular endothelial metabolism, and reduces cardiovascular risk.

### 4.2. Glucagon-like Peptide-1 (GLP-1) Receptor Agonists

GLP-1 receptor agonists are a class of medications that lower blood glucose by mimicking the action of incretin hormones [145]. In addition to effectively controlling blood sugar levels, GLP-1 receptor agonists can reduce insulin dosage and improve postprandial glycemic variability. GLP-1 receptor agonists have also demonstrated potential cardioprotective effects in DCM management [146,147].

GLP-1 receptor agonists exert crucial protective effects in DCM by safeguarding mitochondrial function and modulating oxidative stress pathways [148]. Under the hyperglycemic conditions in DCM pathogenesis, mitochondrial dysfunction and the excessive generation of ROS trigger oxidative stress and inflammatory responses, ultimately resulting in myocardial fibrosis, ventricular remodeling, and impaired cardiac function. GLP-1 receptor agonists such as Exenatide intervene in the progression of DCM through multiple mechanisms, including autophagy activation to clear damaged mitochondria, caspase-1 inhibition to reduce ROS accumulation, and the optimization of energy metabolism via promoted glucose oxidation [149,150,151,152,153].

Exenatide exerts cardioprotective effects at the animal level through a GLP-1 receptor-dependent, multi-target mechanism: it regulates the miR-29b-3p/sarcolemma associated protein (SLMAP) axis to correct abnormal myocardial protein expression and reduce lipid deposition and fibrosis; it simultaneously activates adenosine 5′-monophosphate AMP-activated protein kinase (AMPK)/AKT and inhibits the mammalian target of rapamycin (mTOR) signaling pathways to restore the imbalance in mitochondrial dynamics and autophagy dysfunction [154,155]. This reverses myocardial structural remodeling and cardiac dysfunction in diabetic mice, though its efficacy and safety in clinical patients require further validation [115]. Exendin-4 promotes mitochondrial biogenesis by activating the sirtuin 1 (SIRT1)/PGC-1α/nuclear respiratory factor 1 (NRF-1) pathway, restores the NADPH oxidase 1 (NOX1)/superoxide dismutase (SOD1) antioxidant balance, improves myocardial structural remodeling in diabetic mice, reduces cardiac load markers (atrial natriuretic peptide/brain natriuretic peptide), and inhibits fibrosis (transforming growth factor beta 1), thereby exerting multidimensional cardioprotective effects. However, its efficacy, safety, and mechanism of action in humans require further clinical validation [116,156,157,158,159,160,161,162,163,164,165].

GLP-1 receptor agonists, exemplified by semaglutide, have also shown significant cardiovascular risk reduction. In patients with T2DM and established CVD, semaglutide reduced the risk of major adverse cardiovascular events (MACE) [166]. The recent SELECT (Semaglutide Effects on Cardiovascular Outcomes in People with Overweight or Obesity) trial further demonstrated that semaglutide reduced MACE risk by 20% in adults with overweight or obesity and pre-existing CVD but without diabetes, indicating cardioprotective effects independent of its glucoregulatory actions [167]. These clinical outcomes align with preclinical evidence of GLP-1 receptor agonists-mediated anti-inflammatory, anti-apoptotic, and anti-fibrotic effects in the diabetic heart. Furthermore, the FLOW (Research Study To See How Semaglutide Works Compared to Placebo in People With Type 2 Diabetes and Chronic Kidney Disease) trial revealed that semaglutide significantly reduces the risk of major kidney disease events and cardiovascular outcomes in patients with T2DM and chronic kidney disease, highlighting its organ-protective pleiotropy [168].

Emerging evidence suggests GLP-1 receptor agonists may play an increasingly important role in DCM treatment by restoring mitochondrial function and modulating oxidative stress pathways, becoming an effective strategy for controlling blood sugar, reducing insulin dosage, and improving cardiovascular health.

### 4.3. Mineralocorticoid Receptor Antagonist (MRA)

In DCM, chronic hyperglycemia induces insulin resistance, shifting the myocardial energy substrate’s preference towards free fatty acids. The upregulated cluster of differentiation 36 (CD36) facilitates excessive free fatty acid uptake, activating peroxisome proliferator-activated receptor α (PPARα) and accelerating mitochondrial β-oxidation, which increases mitochondrial reactive oxygen species (mtROS) production and exacerbates myocardial dysfunction. Aldosterone binding to mineralocorticoid receptor (MR) in cardiomyocytes promotes detrimental remodeling. MRAs block cardiac MR, reducing morbidity and mortality in advanced heart failure, with nonsteroidal MRAs like finerenone potentially offering better cardiac protection than traditional steroidal MRAs. Finerenone antagonizes MR, reduces ROS generation, downregulates PPARα and CD36, limits free fatty acids’ entry into mitochondria, and improves ATP/O_2_ coupling efficiency. Furthermore, it modulates mitochondrial dynamics, promotes fusion and biogenesis, reduces oxidative injury, and impedes maladaptive hypertrophy and fibrosis in DCM [121,169,170,171].

Finerenone regulates mitochondrial dynamics by reducing DRP1-S616 phosphorylation and enhancing MFN2 and OPA1 expression, promoting mitochondrial fusion and network integrity. It restores the AMPK-PGC-1α pathway, stimulates mitochondrial biogenesis, and selectively eliminates damaged mitochondria via PINK1/Parkin-mediated mitophagy. Additionally, finerenone augments SIRT3 activity, which activates MnSOD and isocitrate dehydrogenase (IDH2) to detoxify mtROS. Collectively, these mechanisms re-establish mitochondrial quality control, attenuate cardiomyocyte oxidative injury, and impede maladaptive hypertrophy and fibrosis in DCM [172,173]. Finerenone effectively alleviates DCM by antagonizing MR, reducing ROS generation, and optimizing mitochondrial function. Future research should prioritize its long-term effects on DCM progression and its potential in combination therapies to maximize clinical benefits [117].

Eplerenone selectively antagonizes mineralocorticoid receptors to block aldosterone-mediated pathological pathways, providing multi-target protection against DCM: it enhances glutathione (GSH)/SOD ratios and reduces malondialdehyde (MDA) levels to alleviate oxidative stress; downregulates glucose regulated protein78kD (GRP78) and spliced X-box binding protein-1 (XBP1s) to relieve endoplasmic reticulum stress; and inhibits NLRP3 inflammasomes and interleukin-1β (IL-1β) to mitigate inflammation. Its cardioprotective effects are independent of glycemic control, reducing troponin I levels and improving myocardial injury with fewer side effects than spironolactone, offering a new option for combination therapy in DCM [118].

Animal studies demonstrate that spironolactone confers cardioprotection through a tripartite mechanism: mitochondrial restoration (preserving cristae integrity and upregulating ATP synthase F1 subunit alpha (ATP5A1)/cytochrome C oxidase subunit 5B (COX5b)/SIRT1 to enhance bioenergetics), oxidative stress mitigation (activating NRF-1/glutamate–cysteine ligase catalytic subunit (GCLC) antioxidant defenses while suppressing Nox4-driven ROS production), and inflammation–fibrosis suppression (attenuating tumor necrosis factor-α (TNF-α)/monocyte chemoattractant protein-1 (MCP-1) release, macrophage infiltration, and transforming growth factor (TGF-β1)/collagen I deposition). Importantly, these benefits occur independently of glycemic and blood pressure control. Nevertheless, the translation of these preclinical findings to clinical practice warrants validation through large-scale randomized controlled trials [119].

However, the translation of other promising mechanistic targets from preclinical studies to clinical success is not guaranteed, highlighting the complexity of DCM. The ARISE-HF (Aldose Reductase Inhibition for Stabilization of Exercise Capacity in Heart Failure) trial, which evaluated the selective aldose reductase inhibitor AT-001 in patients with DCM and impaired exercise capacity, found that 15 months of treatment did not result in a statistically significant improvement in peak oxygen uptake (VO_2_) compared to placebo [174]. The successful translation of SGLT2 inhibitors and GLP-1 receptor agonists from mechanistic preclinical discovery to proven clinical benefit provides a powerful paradigm. It confirms that interventions targeting core DCM pathophysiological axes—such as metabolic dysregulation, inflammation, and fibrosis—can effectively improve hard clinical endpoints. This bridge from bench to bedside underscores the value of the preclinical research landscape mapped in Table 2. Future therapeutic strategies should focus on combining these foundational therapies with other promising mechanistic approaches (e.g., targeting mitochondrial dysfunction) identified in preclinical studies and employing more clinically representative models (e.g., aged, comorbid) to enhance the predictive validity and translational success of future drug development for DCM.

As a new generation of antidiabetic agents, SGLT2 inhibitors, GLP-1RAs, and finerenone not only provide excellent glycemic control, but also exhibit cardioprotective effects against DCM. These drugs improve mitochondrial function, attenuate oxidative stress and inflammatory responses through various mechanisms, thereby alleviating myocardial damage and improving cardiac function. Future studies will clarify the specific molecular targets in DCM and strengthen the scientific evidence base for clinical application.

## 5. Conclusions

In summary, this article systematically explores the pathogenic link between DCM and mitochondrial homeostasis. Disrupted mitochondrial function and homeostasis contribute to a variety of the pathological features of DCM, including cardiomyocyte apoptosis, inflammatory response, fibrosis, etc., which make mitochondrial homeostasis an important part of DCM’s pathophysiology. Mechanisms such as mitochondrial dynamics, mitochondrial oxidative metabolism, and mitophagy alleviate DCM-related myocardial injury by regulating ATP synthesis, optimizing myocardial energy expenditure caused by impaired mitochondrial decoupling, controlling ROS release from mitochondrial oxidative stress, and removing excess free fatty acids and damaged mitochondria from the heart. Therefore, future investigations into the biological mechanism of mitochondria, particularly through reducing oxidative stress, strengthening free fatty acid metabolism, regulating mitochondrial dynamics and autophagy, and improving mitochondrial oxidative metabolism to restore mitochondrial homeostasis, will provide effective therapeutic approaches to DCM treatment.

## Figures and Tables

**Table 1:** Mitochondrial dysfunction and related mechanisms in diabetic cardiomyopathy.

Mitochondrial Dysfunction	Potential Mechanisms and Related Pathways	Models	Refs.
Mitochondrial oxidative metabolism	Decreased equivalent input, decreased oxidative phosphorylation capacity, increased reactive oxygen species, increased lipid peroxidation	db/db mice	[61]
Mitochondrial oxidative metabolism	Mouse cardiomyocytes under hyperglycemic conditions reduce [Ca^2+^]m and MCU protein levels and induce cardiomyocyte apoptosis	mice; NMCM	[62,63]
Mitochondrial oxidative metabolism	DCM mice exhibit a range of left ventricular mitochondrial changes, including reduced mitochondrial area, increased levels of mitochondrial complex-III and complex-V protein abundance, and reduced oxygen consumption in complex II	mice	[64]
Mitochondrial oxidative metabolism	Increased cellular and mitochondrial fatty acid uptake, increased fatty acid oxidation, decreased LCAD activity and mitochondrial mass	rat	[65]
Mitochondrial oxidative metabolism	Increased O-GlcNAcylation of the transcription factor SP1, many electron transport chain subunits, and other mitochondrial proteins in diabetic mice leads to exacerbated mitochondrial oxidative dysfunction	mice; C_2_C_12_	[66]
Mitochondrial oxidative metabolism	Reduced complex 1 activity, increased H_2_O_2_ and NO production, and decreased ATP production	rat	[67]
Mitochondrial oxidative metabolism	Myocardial ADMA accumulation promotes cardiac and mitochondrial dysfunction in T1DM rats. The underlying mechanism may be related to NOS uncoupling, leading to decreased NO and increased oxidative stress, and ultimately PGC-1α downregulation and UCP2 upregulation.	rat	[68]
Mitochondrial dynamics	Excessive cardiac mitochondrial fission rate in diabetic mice leads to reduced mean mitochondrial size and a reduction in the number of mitochondria per μm^2^; downregulation of MFN2-induced imbalance in mitochondrial dynamics promotes mitochondrial dysfunction and DCM.	db/db mice; NRCM	[69]
Mitochondrial dynamics	High glucose induces DRP1-mediated mitochondrial fission via orai1 calcium channels involved in diabetic cardiomyocyte hypertrophy.	NRCM; rat	[70]
Mitochondrial dynamics	MST1 exacerbates mitochondrial fission, impairs mitochondrial energy metabolism and function, and exacerbates cardiac dysfunction in DCM.	mice; NMCM	[71]
Mitochondrial dynamics	Mitochondrial reactive oxygen species in lipotoxic hearts induce post-translational modifications of AKAP121, DRP1, and OPA1 to promote mitochondrial fission.	NRVC	[72]
Mitophagy	MST1 inhibits mitochondrial autophagy in a SIRT3-Parkin-dependent manner.	mice; NMCM	[73]
Mitophagy	Inhibition of SIRT3-FOXO3A-Parkin signaling-mediated downregulation of mitochondrial autophagy.	mice; NMCM	[74]
Mitophagy	In high-fat-diet-induced DCM, deletion of ATG7 and Parkin leads to impaired mitochondrial autophagy, increased lipid accumulation, exacerbated diastolic dysfunction, and induced systolic dysfunction.	mice	[75]
Mitophagy	Cardiac BRD4 is upregulated in high-fat-diet-induced DCM and inhibits PINK1/Parkin-mediated mitochondrial autophagy, thereby impairing mitochondrial and cardiac structure and function.	mice; NMCM	[76]

Abbreviations: MCU, mitochondrial calcium uniporter; NMCM, neonatal mouse cardiac myocyte; NRVC, neonatal rat ventricular cardiomyocyte; LCAD, Long chain acyl-CoA dehydrogenase deficiency; SP1, Specificity protein 1; ADMA, asymmetric dimethylarginine; Parkin, Parkin RBR E3 ubiquitin protein ligase; UCP2, uncoupling protein 2; NOS, nitric oxide synthase; MST1, macrophage stimulating 1; SIRT3, sirtuin 3; FOXO3A, Forkhead box O3A; ATG7, autophagy-related 7 gene; BRD4, bromodomain containing 4; PINK1, PTEN-induced putative kinase 1.

**Table 2:** Drugs targeting mitochondrial homeostasis for diabetic cardiomyopathy.

Protective Drugs	Target	Therapeutic Mechanisms	Models	Dose	Intervention Duration	Number	Author, Year	Refs.
Empagliflozin	ROS	Suppressing mitochondrial ROS generation, activating the NRF2/antioxidant enzyme axis, and reducing oxidative damage markers.	db/db mice (oral)	10 mg/kg/day	8 w	10	Weijuan Cai et al., 2024	[110]
Dapagliflozin	Fibrosis	Left ventricular remodeling reversal → improved cardiac diastolic/systolic function → reduced myocardial collagen deposition.	SD rat (oral)	1 mg/kg/day	8 w	15	Jingjing Tian et al., 2021	[111]
Canagliflozin	Mitophagy	PINK1-Parkin ↑ → mitochondrial autophagy ↑ → restoration of mitochondrial structure and function → improved cardiac function.	Mice (oral)	30 mg/kg/d	12 w	12	Chunru Yang et al., 2024	[112]
Pioglitazone	Mitochondrial oxidative metabolism; Mitochondrial dynamics	Activate the PPAR-γ/PGC-1α pathway to restore mitochondrial biogenesis (NRF1/TFAM) and fusion-fission homeostasis (OPA1/MFN1/DRP1), thereby improving mitochondrial structure and function, alleviating oxidative stress and inflammation, reversing diabetes-induced atrial structural and electrical remodeling, and preventing atrial fibrillation.	Rabbit	4 mg/kg/d	8 w	8	Zhiwei Zhang et al., 2021	[113]
Liraglutide	Ferroptosis	Activation of the NRF2 pathway upregulates ferroptosis-inhibiting proteins such as GPX4 and FTH-1, reducing myocardial iron deposition and lipid peroxidation (MDA ↓, GSH ↑). This inhibits ferroptosis, improves mitochondrial morphology and function, and alleviates cardiac remodeling and dysfunction in DCM.	The spontaneously diabetic Goto-Kakizaki rat (subcutaneous injections)	200 μg/kg/d	8 w	15	Xuepin Chen et al., 2025	[114]
Exenatide	Fibrosis	Lipid deposition ↓; myocardial fibrosis ↓; BNP ↓; SLMAP ↓	Mice (intraperitoneal injection)	10 mg/kg/w	12 w	12	Ping Fang et al., 2023	[115]
Exendin-4	Mitochondrial oxidative metabolism	SIRT1-PGC-1α-NRF-1 axis: SIRT1 deacetylation activates PGC-1α, promoting mitochondrial biogenesis and improving energy metabolism.	Mice (intraperitoneal injection)	1 nmol/kg	8 w	8	Yingying Cai et al., 2018	[116]
Finerenone	ROS; Ferroptosis	ROS ↓; ATP ↑; improved left ventricular systolic pressure and diastolic dysfunction; reduced myocardial fibrosis and apoptosis.	Rat (intraperitoneal injection)	1 mg/kg/day	8 w	6	Tao Jin et al., 2023	[117]
Eplerenone	Mitochondrial oxidative metabolism	Improved oxidative stress (↑ GSH/SOD, ↓ MDA); alleviated endoplasmic reticulum stress (↓ GRP78, ↓ XBP1s); reduced inflammation (↓ NLRP3, ↓ IL-1β); improved myocardial injury (↓ Troponin I).	Rat (intraperitoneal injection)	100 mg/kg/day	8 w	8	Hamid Yaghooti et al., 2025	[118]
Spironolactone	Mitochondrial oxidative metabolism; ROS	ATP5A1/COX5b/SIRT1 ↑; activate the NRF-1/GCLC antioxidant pathway and inhibit NOX4; suppress inflammation and fibrosis.	SD Rat (gavage)	20 mg/kg/day	12 w	8	Wenjuan Liu et al., 2018	[119]
Empagliflozin	ROS	Restore mitochondrial ketone metabolism → activate NRF2 antioxidant defense → upregulate BCL-2 to maintain membrane integrity.	H9c2	5 μM	48 h	/	Weijuan Cai et al., 2024	[110]
Dapagliflozin	Fibrotic	Suppressing EndMT and fibroblast activation via AMPKα/TGF-β/Smad signaling.	HUVEC	1 μM	48 h	/	Jingjing Tian et al., 2021	[111]
Canagliflozin	Mitophagy	AMPK ↑ → PINK1-Parkin ↑ → mitochondrial autophagy ↑ → mitochondrial function restoration.	H9c2	20 μM	48 h	/	Chunru Yang et al., 2024	[112]
Pioglitazone	ROS	ROS ↓; MMP ↑; PPAR-γ/PGC-1α ↑.	HL-1	10 μM	1 h	/	Zhiwei Zhang et al., 2021	[113]
Liraglutide	Ferroptosis	Antioxidant and anti-ferroptotic proteins (GPX4, FTH-1), reduction in ferroptotic markers (PTGS2, Fe^2+^, lipid peroxides).	H9c2	200 nM	24 h	/	Xuepin Chen et al., 2025	[114]
Exendin-4	Mitophagy	GLP-1, AMPK-autophagy ↑, mTOR signaling ↓, mitochondrial fusion ↑, excessive fission ↓, antioxidant ↑, mitochondrial autophagy ↑, myocardial oxidative stress ↓, mitochondrial dysfunction ↓.	H9c2	5–100 nM		/	Warisara Parichatikanond et al., 2024	[120]
Finerenone	ROS; Ferroptosis	GRK5 ↑; aldosterone ↓; apoptosis ↓; oxidative stress ↓; fibrosis ↓.	H9c2	10 mM	24 h	/	Pollard CM et al., 2022	[121]
Spironolactone	ROS	ROS ↓	H9c2	0.1 μM	72 h	/	Wenjuan Liu et al., 2018	[119]

Note: ↑, upregulation; ↓, downregulation; →, direction of change/influence; NRF2, nuclear factor erythroid 2-related factor 2; db/db mice, diabetes mouse; NRF1, nuclear respiratory factor 1; TFAM, transcription factor A, mitochondrial; GPX4, glutathione peroxidase 4; MDA, malondialdehyde; GSH, glutathione; BNP, B-type natriuretic; SLMAP, sarcolemma-associated protein; SIRT1, sirtuin 1; PGC-1α, peroxisome proliferator-activated receptor γ coactivator 1-alpha; GRP78, glucose regulated protein78kD; XBP1s, spliced X-box binding protein-1; NLRP3, NOD-like receptor family pyrin domain containing 3; IL-1β, interleukin-1 beta; ATP5A, ATP synthase F1 subunit alpha; COX5b, cytochrome c oxidase subunit 5B; GCLC, glutamate–cysteine ligase catalytic subunit; NOX4, NADPH oxidase 4; BCL-2, BCL2 apoptosis regulator; EndMT, endothelial-to-mesenchymal transition; AMPK, adenosine 5‘-monophosphate (AMP)-activated protein kinase; AMPKα, protein kinase AMP-activated catalytic subunit alpha 2; TGF-β, transforming growth factor-β; PTGS2, prostaglandin–endoperoxide synthase 2; GLP-1, glucagon-like peptide-1; MMP, mitochondrial membrane potential; mTOR, mammalian target of rapamycin; GRK5, G protein-coupled receptor kinase 5.

## Data Availability

No new data were created or analyzed in this study. Data sharing is not applicable to this article.

## Abbreviations

The following abbreviations are used in this manuscript:

AMPK	AMP-activated protein kinase
AGO2	Argonaute2
ADMA	Asymmetric Dimethylarginine
AKAP121	A-kinase anchor protein 121
ATP	Adenosine triphosphate
ATP5A1	ATP synthase F1 subunit alpha
ADP	Adenosine diphosphate
AKT	Protein kinase B
BNIP3	BCL2/adenovirus E1B 19 kDa interacting protein 3
BDH1	3-hydroxybutyrate dehydrogenase 1
BRD4	Bromodomain protein 4
CVD	Cardiovascular disease
COX5b	Cytochrome C oxidase subunit 5B
CD36	Cluster of differentiation 36
CDK1	Cyclin-dependent kinases
CYTB	Cytochrome b
DCM	Diabetic cardiomyopathy
Drp1	Dynamin-related protein 1
ERα	Estrogen receptor α
EndMT	Endothelial-to-mesenchymal transition
Empa	Empagliflozin
eNOS	Endothelial nitric oxide synthase
ETC	Electron transport chain
FIS1	Fission1
FGF21	Fibroblast growth factor 21
FUNDC1	FUN14 domain containing 1
FOXO3	Forkhead box O3
GTPase	GTP hydrolysis
GCLC	Glutamate–cysteine ligase catalytic subunit
GABARAP	Gamma–aminobutyric acid receptor-associated protein
GPX4	Glutathione Peroxidase 4
GRP78	Glucose-regulated protein78kD
GSH	Glutathione
GLP-1RAs	GLP-1 receptor agonists
GTP	Guanosine triphosphate
HF	Heart failure
HFpEF	Heart failure and preserved ejection fraction
HFrEF	Heart failure and reduced ejection fraction
HO-1	Heme oxygenase-1
IMM	Inner mitochondrial membrane
IDH2	Isocitrate dehydrogenase
LCAD	Long-chain acyl-CoA dehydrogenase deficiency
LC3	Microtubule-associated protein 1 light chain 3
LIR	LC3 interaction region
mTOR	Mammalian target of rapamycin
MACE	Major adverse cardiovascular events
MDA	Malondialdehyde
MICU1	Mitochondrial calcium uptake 1
MCU	Mitochondrial calcium uniporter
MR	Mineralocorticoid receptor
MRA	Mineralocorticoid receptor antagonists
MMP	Mitochondrial membrane potential
MFN1	Mitofusin 1
MFF	Mitochondrial fission factor
MiD51	Mitochondrial dynamics proteins of 51 kDa
MiD49	Mitochondrial dynamics proteins of 49 kDa
MTFP1	Mitochondrial fission process 1
MFN2	Mitofusin 2
MDVs	Mitochondria-derived vesicles
MnSOD	Mn superoxide dismutase
NHE-1	Sodium–hydrogen exchanger 1
NLRP3	NOD-like receptor family pyrin domain containing
NIX	NIP3-like protein X
NO	Nitric oxide
NMCMs	Neonatal Mouse Cardiac Myocytes
NRCMs	Neonatal Rat Cardiomyocytes
NRVCs	Neonatal Rat Ventricular Cardiomyocytes
NADPH	Nicotinamide adenine dinucleotide phosphate
NRF-1	Nuclear Respiratory Factor 1
NRF-2	Nuclear Factor erythroid-derived 2-like 2
NF-κB	Nuclear Factor kappa-light-chain-enhancer of activated B cells
Nox4	NADPH oxidase 4
NGR1	Notoginsenoside R1
OPA1	Optic Atrophy 1
OMM	Outer mitochondrial membrane
OMA1	Overlapping with the M-AAA protease 1 homolog
OXCT1	3-Oxoacid CoA-Transferase 1
PPA2	Pyrophosphatase phosphatase 2A
PD	Parkinson’s disease
PINK1	PTEN-induced putative kinase 1
Parkin	E3 ubiquitin–protein ligase parkin
PGC-1α	Peroxisome proliferator-activated receptor gamma coactivator 1-alpha
PPAR-γ	Peroxisome proliferator-activated receptor gamma
ROS	Reactive oxygen species
SLMAP	Sarcolemma-associated protein
SIRT1	Sirtuin 1
SMEM	Small MTFP1-enriched mitochondria
SGLT2	Sodium–glucose co-transporter 2
SGLT2i	Sodium–glucose cotransporter-2 inhibitors
SP1	Specificity Protein 1
SIRT3	Sirtuin 3
SOD	Super Oxide Dimutese
TZD	Thiazolidinedione
TFAM	Mitochondrial transcription factor A
TCA	Tricarboxylic acid
T2DM	Type 2 diabetes mellitus
T1DM	Type 1 diabetes mellitus
TRPV1	Transient receptor potential vanilloid 1
UCP2	Uncoupling Protein 2
XBP1s	X-box binding protein-1

## References

[1] Liu L., Zhang J., Cheng Y., Zhu M., Xiao Z., Ruan G., Wei Y. (2022). Gut microbiota: A new target for T2DM prevention and treatment. Front. Endocrinol..

[2] Jaacks L.M., Siegel K.R., Gujral U.P., Narayan K.M. (2016). Type 2 diabetes: A 21st century epidemic. Best Pract. Res. Clin. Endocrinol. Metab..

[3] American Diabetes Association (2014). Diagnosis and classification of diabetes mellitus. Diabetes Care.

[4] Sung K.C., Lee M.Y., Kim Y.H., Huh J.H., Kim J.Y., Wild S.H., Byrne C.D. (2018). Obesity and incidence of diabetes: Effect of absence of metabolic syndrome, insulin resistance, inflammation and fatty liver. Atherosclerosis.

[5] Ong K.L., Stafford L.K., McLaughlin S.A., Boyko E.J., Vollset S.E., Smith A.E., Dalton B.E., Duprey J., Cruz J.A., Hagins H. (2023). Global, regional, and national burden of diabetes from 1990 to 2021, with projections of prevalence to 2050: A systematic analysis for the Global Burden of Disease Study 2021. Lancet.

[6] Zimmet P., Alberti K.G., Shaw J. (2001). Global and societal implications of the diabetes epidemic. Nature.

[7] Deshpande A.D., Harris-Hayes M., Schootman M. (2008). Epidemiology of diabetes and diabetes-related complications. Phys. Ther..

[8] Farmaki P., Damaskos C., Garmpis N., Garmpi A., Savvanis S., Diamantis E. (2020). Complications of the Type 2 Diabetes Mellitus. Curr. Cardiol. Rev..

[9] Jaacks L.M., Vandevijvere S., Pan A., McGowan C.J., Wallace C., Imamura F., Mozaffarian D., Swinburn B., Ezzati M. (2019). The obesity transition: Stages of the global epidemic. Lancet Diabetes Endocrinol..

[10] Younossi Z.M., Koenig A.B., Abdelatif D., Fazel Y., Henry L., Wymer M. (2016). Global epidemiology of nonalcoholic fatty liver disease-Meta-analytic assessment of prevalence, incidence, and outcomes. Hepatology.

[11] Tomic D., Shaw J.E., Magliano D.J. (2022). The burden and risks of emerging complications of diabetes mellitus. Nat. Rev. Endocrinol..

[12] Lu Y., Lu Y., Meng J., Wang Z. (2021). Pyroptosis and Its Regulation in Diabetic Cardiomyopathy. Front. Physiol..

[13] Paolillo S., Marsico F., Prastaro M., Renga F., Esposito L., De Martino F., Di Napoli P., Esposito I., Ambrosio A., Ianniruberto M. (2019). Diabetic Cardiomyopathy: Definition, Diagnosis, and Therapeutic Implications. Heart Fail. Clin..

[14] Jia G., Hill M.A., Sowers J.R. (2018). Diabetic Cardiomyopathy: An Update of Mechanisms Contributing to This Clinical Entity. Circ. Res..

[15] Yilmaz S., Canpolat U., Aydogdu S., Abboud H.E. (2015). Diabetic Cardiomyopathy; Summary of 41 Years. Korean Circ. J..

[16] Dannenberg L., Weske S., Kelm M., Levkau B., Polzin A. (2021). Cellular mechanisms and recommended drug-based therapeutic options in diabetic cardiomyopathy. Pharmacol. Ther..

[17] Donath M.Y., Dinarello C.A., Mandrup-Poulsen T. (2019). Targeting innate immune mediators in type 1 and type 2 diabetes. Nat. Rev. Immunol..

[18] Kenny H.C., Abel E.D. (2019). Heart Failure in Type 2 Diabetes Mellitus. Circ. Res..

[19] Shi X., Liu C., Chen J., Zhou S., Li Y., Zhao X., Xing J., Xue J., Liu F., Li F. (2023). Endothelial MICU1 alleviates diabetic cardiomyopathy by attenuating nitrative stress-mediated cardiac microvascular injury. Cardiovasc. Diabetol..

[20] Batista J.P.T., Faria A.O.V., Ribeiro T.F.S., Simões E.S.A.C. (2023). The Role of Renin-Angiotensin System in Diabetic Cardiomyopathy: A Narrative Review. Life.

[21] Salvatore T., Galiero R., Caturano A., Vetrano E., Rinaldi L., Coviello F., Di Martino A., Albanese G., Colantuoni S., Medicamento G. (2022). Dysregulated Epicardial Adipose Tissue as a Risk Factor and Potential Therapeutic Target of Heart Failure with Preserved Ejection Fraction in Diabetes. Biomolecules.

[22] Vasileiou P.V.S., Evangelou K., Vlasis K., Fildisis G., Panayiotidis M.I., Chronopoulos E., Passias P.G., Kouloukoussa M., Gorgoulis V.G., Havaki S. (2019). Mitochondrial Homeostasis and Cellular Senescence. Cells.

[23] Li X., Jiang O., Chen M., Wang S. (2024). Mitochondrial homeostasis: Shaping health and disease. Curr. Med..

[24] Nowinski S.M., Solmonson A., Rusin S.F., Maschek J.A., Bensard C.L., Fogarty S., Jeong M.Y., Lettlova S., Berg J.A., Morgan J.T. (2020). Mitochondrial fatty acid synthesis coordinates oxidative metabolism in mammalian mitochondria. eLife.

[25] Martínez-Reyes I., Chandel N.S. (2020). Mitochondrial TCA cycle metabolites control physiology and disease. Nat. Commun..

[26] Nolfi-Donegan D., Braganza A., Shiva S. (2020). Mitochondrial electron transport chain: Oxidative phosphorylation, oxidant production, and methods of measurement. Redox Biol..

[27] Zhao R.Z., Jiang S., Zhang L., Yu Z.B. (2019). Mitochondrial electron transport chain, ROS generation and uncoupling (Review). Int. J. Mol. Med..

[28] Bhat A.H., Dar K.B., Anees S., Zargar M.A., Masood A., Sofi M.A., Ganie S.A. (2015). Oxidative stress, mitochondrial dysfunction and neurodegenerative diseases; a mechanistic insight. Biomed. Pharmacother..

[29] Zong Y., Li H., Liao P., Chen L., Pan Y., Zheng Y., Zhang C., Liu D., Zheng M., Gao J. (2024). Mitochondrial dysfunction: Mechanisms and advances in therapy. Signal Transduct. Target. Ther..

[30] Quirós P.M., Langer T., López-Otín C. (2015). New roles for mitochondrial proteases in health, ageing and disease. Nat. Rev. Mol. Cell Biol..

[31] Chen W., Zhao H., Li Y. (2023). Mitochondrial dynamics in health and disease: Mechanisms and potential targets. Signal Transduct. Target. Ther..

[32] Adebayo M., Singh S., Singh A.P., Dasgupta S. (2021). Mitochondrial fusion and fission: The fine-tune balance for cellular homeostasis. FASEB J..

[33] Bleazard W., McCaffery J.M., King E.J., Bale S., Mozdy A., Tieu Q., Nunnari J., Shaw J.M. (1999). The dynamin-related GTPase Dnm1 regulates mitochondrial fission in yeast. Nat. Cell Biol..

[34] Ji W.K., Hatch A.L., Merrill R.A., Strack S., Higgs H.N. (2015). Actin filaments target the oligomeric maturation of the dynamin GTPase Drp1 to mitochondrial fission sites. eLife.

[35] Losón O.C., Song Z., Chen H., Chan D.C. (2013). Fis1, Mff, MiD49, and MiD51 mediate Drp1 recruitment in mitochondrial fission. Mol. Biol. Cell.

[36] Otera H., Wang C., Cleland M.M., Setoguchi K., Yokota S., Youle R.J., Mihara K. (2010). Mff is an essential factor for mitochondrial recruitment of Drp1 during mitochondrial fission in mammalian cells. J. Cell Biol..

[37] Mishra S.R., Mishra P., Mahapatra K.K., Behera B.P., Kendre G., Alotaibi M.R., Pandey V., Patro B.S., Klionsky D.J., Bhutia S.K. (2026). PPA2 activates MTFP1-DNM1L fission signaling to govern mitochondrial proliferation and mitophagy. Autophagy.

[38] Tábara L.C., Burr S.P., Frison M., Chowdhury S.R., Paupe V., Nie Y., Johnson M., Villar-Azpillaga J., Viegas F., Segawa M. (2024). MTFP1 controls mitochondrial fusion to regulate inner membrane quality control and maintain mtDNA levels. Cell.

[39] Tilokani L., Nagashima S., Paupe V., Prudent J. (2018). Mitochondrial dynamics: Overview of molecular mechanisms. Essays Biochem..

[40] Chan D.C. (2006). Mitochondria: Dynamic organelles in disease, aging, and development. Cell.

[41] Koshiba T., Detmer S.A., Kaiser J.T., Chen H., McCaffery J.M., Chan D.C. (2004). Structural basis of mitochondrial tethering by mitofusin complexes. Science.

[42] Cao Y.L., Meng S., Chen Y., Feng J.X., Gu D.D., Yu B., Li Y.J., Yang J.Y., Liao S., Chan D.C. (2017). MFN1 structures reveal nucleotide-triggered dimerization critical for mitochondrial fusion. Nature.

[43] Griparic L., van der Wel N.N., Orozco I.J., Peters P.J., van der Bliek A.M. (2004). Loss of the intermembrane space protein Mgm1/OPA1 induces swelling and localized constrictions along the lengths of mitochondria. J. Biol. Chem..

[44] Yamada T., Ikeda A., Murata D., Wang H., Zhang C., Khare P., Adachi Y., Ito F., Quirós P.M., Blackshaw S. (2025). Dual regulation of mitochondrial fusion by Parkin-PINK1 and OMA1. Nature.

[45] Villa E., Proïcs E., Rubio-Patiño C., Obba S., Zunino B., Bossowski J.P., Rozier R.M., Chiche J., Mondragón L., Riley J.S. (2017). Parkin-Independent Mitophagy Controls Chemotherapeutic Response in Cancer Cells. Cell Rep..

[46] Matsuda N., Sato S., Shiba K., Okatsu K., Saisho K., Gautier C.A., Sou Y.S., Saiki S., Kawajiri S., Sato F. (2010). PINK1 stabilized by mitochondrial depolarization recruits Parkin to damaged mitochondria and activates latent Parkin for mitophagy. J. Cell Biol..

[47] Koyano F., Okatsu K., Kosako H., Tamura Y., Go E., Kimura M., Kimura Y., Tsuchiya H., Yoshihara H., Hirokawa T. (2014). Ubiquitin is phosphorylated by PINK1 to activate parkin. Nature.

[48] Geisler S., Holmström K.M., Skujat D., Fiesel F.C., Rothfuss O.C., Kahle P.J., Springer W. (2010). PINK1/Parkin-mediated mitophagy is dependent on VDAC1 and p62/SQSTM1. Nat. Cell Biol..

[49] McLelland G.L., Soubannier V., Chen C.X., McBride H.M., Fon E.A. (2014). Parkin and PINK1 function in a vesicular trafficking pathway regulating mitochondrial quality control. EMBO J..

[50] Schweers R.L., Zhang J., Randall M.S., Loyd M.R., Li W., Dorsey F.C., Kundu M., Opferman J.T., Cleveland J.L., Miller J.L. (2007). NIX is required for programmed mitochondrial clearance during reticulocyte maturation. Proc. Natl. Acad. Sci. USA.

[51] Liu K., Zhao Q., Sun H., Liu L., Wang C., Li Z., Xu Y., Wang L., Zhang L., Zhang H. (2022). BNIP3 (BCL2 interacting protein 3) regulates pluripotency by modulating mitochondrial homeostasis via mitophagy. Cell Death Dis..

[52] Novak I., Dikic I. (2011). Autophagy receptors in developmental clearance of mitochondria. Autophagy.

[53] Chen G., Han Z., Feng D., Chen Y., Chen L., Wu H., Huang L., Zhou C., Cai X., Fu C. (2014). A regulatory signaling loop comprising the PGAM5 phosphatase and CK2 controls receptor-mediated mitophagy. Mol. Cell.

[54] Liu L., Li Y., Chen Q. (2021). The Emerging Role of FUNDC1-Mediated Mitophagy in Cardiovascular Diseases. Front. Physiol..

[55] Chen Z., Liu L., Cheng Q., Li Y., Wu H., Zhang W., Wang Y., Sehgal S.A., Siraj S., Wang X. (2017). Mitochondrial E3 ligase MARCH5 regulates FUNDC1 to fine-tune hypoxic mitophagy. EMBO Rep..

[56] Ferrucci L., Guerra F., Bucci C., Marzetti E., Picca A. (2024). Mitochondria break free: Mitochondria-derived vesicles in aging and associated conditions. Ageing Res. Rev..

[57] König T., Nolte H., Aaltonen M.J., Tatsuta T., Krols M., Stroh T., Langer T., McBride H.M. (2021). MIROs and DRP1 drive mitochondrial-derived vesicle biogenesis and promote quality control. Nat. Cell Biol..

[58] König T., McBride H.M. (2024). Mitochondrial-derived vesicles in metabolism, disease, and aging. Cell Metab..

[59] Ryan T.A., Phillips E.O., Collier C.L., Robinson A.J., Routledge D., Wood R.E., Assar E.A., Tumbarello D.A. (2020). Tollip coordinates Parkin-dependent trafficking of mitochondrial-derived vesicles. EMBO J..

[60] Peng T., Xie Y., Sheng H., Wang C., Lian Y., Xie N. (2022). Mitochondrial-derived vesicles: Gatekeepers of mitochondrial response to oxidative stress. Free Radic. Biol. Med..

[61] Boudina S., Sena S., Theobald H., Sheng X., Wright J.J., Hu X.X., Aziz S., Johnson J.I., Bugger H., Zaha V.G. (2007). Mitochondrial energetics in the heart in obesity-related diabetes: Direct evidence for increased uncoupled respiration and activation of uncoupling proteins. Diabetes.

[62] Ji L., Liu F., Jing Z., Huang Q., Zhao Y., Cao H., Li J., Yin C., Xing J., Li F. (2017). MICU1 Alleviates Diabetic Cardiomyopathy Through Mitochondrial Ca(2+)-Dependent Antioxidant Response. Diabetes.

[63] Diaz-Juarez J., Suarez J., Cividini F., Scott B.T., Diemer T., Dai A., Dillmann W.H. (2016). Expression of the mitochondrial calcium uniporter in cardiac myocytes improves impaired mitochondrial calcium handling and metabolism in simulated hyperglycemia. Am. J. Physiol. Cell Physiol..

[64] Parker A.M., Tate M., Prakoso D., Deo M., Willis A.M., Nash D.M., Donner D.G., Crawford S., Kiriazis H., Granata C. (2021). Characterisation of the Myocardial Mitochondria Structural and Functional Phenotype in a Murine Model of Diabetic Cardiomyopathy. Front. Physiol..

[65] Lou P.H., Lucchinetti E., Scott K.Y., Huang Y., Gandhi M., Hersberger M., Clanachan A.S., Lemieux H., Zaugg M. (2017). Alterations in fatty acid metabolism and sirtuin signaling characterize early type-2 diabetic hearts of fructose-fed rats. Physiol. Rep..

[66] Wende A.R., Schell J.C., Ha C.M., Pepin M.E., Khalimonchuk O., Schwertz H., Pereira R.O., Brahma M.K., Tuinei J., Contreras-Ferrat A. (2020). Maintaining Myocardial Glucose Utilization in Diabetic Cardiomyopathy Accelerates Mitochondrial Dysfunction. Diabetes.

[67] Rukavina-Mikusic I.A., Rey M., Martinefski M., Tripodi V., Valdez L.B. (2021). Temporal evolution of cardiac mitochondrial dysfunction in a type 1 diabetes model. Mitochondrial complex I impairment, and H(2)O(2) and NO productions as early subcellular events. Free Radic. Biol. Med..

[68] Xiong Y., He Y.L., Li X.M., Nie F., Zhou X.K. (2021). Endogenous asymmetric dimethylarginine accumulation precipitates the cardiac and mitochondrial dysfunctions in type 1 diabetic rats. Eur. J. Pharmacol..

[69] Hu L., Ding M., Tang D., Gao E., Li C., Wang K., Qi B., Qiu J., Zhao H., Chang P. (2019). Targeting mitochondrial dynamics by regulating Mfn2 for therapeutic intervention in diabetic cardiomyopathy. Theranostics.

[70] Wu Q.R., Zheng D.L., Liu P.M., Yang H., Li L.A., Kuang S.J., Lai Y.Y., Rao F., Xue Y.M., Lin J.J. (2021). High glucose induces Drp1-mediated mitochondrial fission via the Orai1 calcium channel to participate in diabetic cardiomyocyte hypertrophy. Cell Death Dis..

[71] Feng X., Wang S., Yang X., Lin J., Man W., Dong Y., Zhang Y., Zhao Z., Wang H., Sun D. (2020). Mst1 Knockout Alleviates Mitochondrial Fission and Mitigates Left Ventricular Remodeling in the Development of Diabetic Cardiomyopathy. Front. Cell Dev. Biol..

[72] Tsushima K., Bugger H., Wende A.R., Soto J., Jenson G.A., Tor A.R., McGlauflin R., Kenny H.C., Zhang Y., Souvenir R. (2018). Mitochondrial Reactive Oxygen Species in Lipotoxic Hearts Induce Post-Translational Modifications of AKAP121, DRP1, and OPA1 That Promote Mitochondrial Fission. Circ. Res..

[73] Wang S., Zhao Z., Fan Y., Zhang M., Feng X., Lin J., Hu J., Cheng Z., Sun C., Liu T. (2019). Mst1 inhibits Sirt3 expression and contributes to diabetic cardiomyopathy through inhibiting Parkin-dependent mitophagy. Biochim. Biophys. Acta Mol. Basis Dis..

[74] Yu W., Gao B., Li N., Wang J., Qiu C., Zhang G., Liu M., Zhang R., Li C., Ji G. (2017). Sirt3 deficiency exacerbates diabetic cardiac dysfunction: Role of Foxo3A-Parkin-mediated mitophagy. Biochim. Biophys. Acta Mol. Basis Dis..

[75] Tong M., Saito T., Zhai P., Oka S.I., Mizushima W., Nakamura M., Ikeda S., Shirakabe A., Sadoshima J. (2019). Mitophagy Is Essential for Maintaining Cardiac Function During High Fat Diet-Induced Diabetic Cardiomyopathy. Circ. Res..

[76] Mu J., Zhang D., Tian Y., Xie Z., Zou M.H. (2020). BRD4 inhibition by JQ1 prevents high-fat diet-induced diabetic cardiomyopathy by activating PINK1/Parkin-mediated mitophagy in vivo. J. Mol. Cell. Cardiol..

[77] Nguyen B.Y., Ruiz-Velasco A., Bui T., Collins L., Wang X., Liu W. (2019). Mitochondrial function in the heart: The insight into mechanisms and therapeutic potentials. Br. J. Pharmacol..

[78] Dia M., Gomez L., Thibault H., Tessier N., Leon C., Chouabe C., Ducreux S., Gallo-Bona N., Tubbs E., Bendridi N. (2020). Reduced reticulum-mitochondria Ca(2+) transfer is an early and reversible trigger of mitochondrial dysfunctions in diabetic cardiomyopathy. Basic Res. Cardiol..

[79] Jaquenod De Giusti C., Palomeque J., Mattiazzi A. (2022). Ca(2+) mishandling and mitochondrial dysfunction: A converging road to prediabetic and diabetic cardiomyopathy. Pflug. Arch..

[80] Li X., Hou J., Du J., Feng J., Yang Y., Shen Y., Chen S., Feng J., Yang D., Li D. (2018). Potential Protective Mechanism in the Cardiac Microvascular Injury. Hypertension.

[81] Nakamura H., Matoba S., Iwai-Kanai E., Kimata M., Hoshino A., Nakaoka M., Katamura M., Okawa Y., Ariyoshi M., Mita Y. (2012). p53 promotes cardiac dysfunction in diabetic mellitus caused by excessive mitochondrial respiration-mediated reactive oxygen species generation and lipid accumulation. Circ. Heart Fail..

[82] Berthiaume J.M., Kurdys J.G., Muntean D.M., Rosca M.G. (2019). Mitochondrial NAD(+)/NADH Redox State and Diabetic Cardiomyopathy. Antioxid. Redox Signal..

[83] Lopaschuk G.D., Karwi Q.G., Tian R., Wende A.R., Dale E. (2021). Cardiac Energy Metabolism in Heart Failure. Circ. Res..

[84] Qi B., He L., Zhao Y., Zhang L., He Y., Li J., Li C., Zhang B., Huang Q., Xing J. (2020). Akap1 deficiency exacerbates diabetic cardiomyopathy in mice by NDUFS1-mediated mitochondrial dysfunction and apoptosis. Diabetologia.

[85] Sciarretta S., Maejima Y., Zablocki D., Sadoshima J. (2018). The Role of Autophagy in the Heart. Annu. Rev. Physiol..

[86] Zhang H., Muhetarijiang M., Chen R.J., Hu X., Han J., Zheng L., Chen T. (2024). Mitochondrial Dysfunction: A Roadmap for Understanding and Tackling Cardiovascular Aging. Aging Dis..

[87] Hao Y., Liu W. (2023). Metabolic Changes in Cardiac Aging. Rev. Cardiovasc. Med..

[88] Patyal P., Azhar G., Verma A., Sharma S., Shrivastava J., Abdi S.A.H., Zhang X., Wei J.Y. (2025). Mitochondrial Dynamics in Aging Heart. Biomedicines.

[89] Rudokas M.W., McKay M., Toksoy Z., Eisen J.N., Bögner M., Young L.H., Akar F.G. (2024). Mitochondrial network remodeling of the diabetic heart: Implications to ischemia related cardiac dysfunction. Cardiovasc. Diabetol..

[90] Peng C., Zhang Y., Lang X., Zhang Y. (2023). Role of mitochondrial metabolic disorder and immune infiltration in diabetic cardiomyopathy: New insights from bioinformatics analysis. J. Transl. Med..

[91] Werbner B., Tavakoli-Rouzbehani O.M., Fatahian A.N., Boudina S. (2023). The dynamic interplay between cardiac mitochondrial health and myocardial structural remodeling in metabolic heart disease, aging, and heart failure. J. Cardiovasc. Aging.

[92] Whitley B.N., Engelhart E.A., Hoppins S. (2019). Mitochondrial dynamics and their potential as a therapeutic target. Mitochondrion.

[93] Dai W., Jiang L. (2019). Dysregulated Mitochondrial Dynamics and Metabolism in Obesity, Diabetes, and Cancer. Front. Endocrinol..

[94] Lin J., Duan J., Wang Q., Xu S., Zhou S., Yao K. (2022). Mitochondrial Dynamics and Mitophagy in Cardiometabolic Disease. Front. Cardiovasc. Med..

[95] Ding M., Feng N., Tang D., Feng J., Li Z., Jia M., Liu Z., Gu X., Wang Y., Fu F. (2018). Melatonin prevents Drp1-mediated mitochondrial fission in diabetic hearts through SIRT1-PGC1α pathway. J. Pineal Res..

[96] Li W., Ji L., Tian J., Tang W., Shan X., Zhao P., Chen H., Zhang C., Xu M., Lu R. (2021). Ophiopogonin D alleviates diabetic myocardial injuries by regulating mitochondrial dynamics. J. Ethnopharmacol..

[97] Zhang T., Li Z., Xu Y., Xu C., Wang H., Rui T. (2025). Regulation of mitochondrial dynamics in cardiomyocytes: Implications for cardiac health and disease. Front. Cell Dev. Biol..

[98] Muthu S., Tran Z., Thilagavathi J., Bolarum T., Azzam E.I., Suzuki C.K., Sundararajan V. (2025). Aging triggers mitochondrial, endoplasmic reticulum, and metabolic stress responses in the heart. J. Cardiovasc. Aging.

[99] Zhi F., Pu X., Wei W., Liu L., Liu C., Chen Y., Chang X., Xu H. (2024). Modulating mitochondrial dynamics ameliorates left ventricular dysfunction by suppressing diverse cell death pathways after diabetic cardiomyopathy. Int. J. Med. Sci..

[100] Zhao G.J., Zhao C.L., Ouyang S., Deng K.Q., Zhu L., Montezano A.C., Zhang C., Hu F., Zhu X.Y., Tian S. (2020). Ca^2+^-Dependent NOX5 (NADPH Oxidase 5) Exaggerates Cardiac Hypertrophy Through Reactive Oxygen Species Production. Hypertension.

[101] Zheng H., Zhu H., Liu X., Huang X., Huang A., Huang Y. (2021). Mitophagy in Diabetic Cardiomyopathy: Roles and Mechanisms. Front. Cell Dev. Biol..

[102] Yang F., Qin Y., Wang Y., Meng S., Xian H., Che H., Lv J., Li Y., Yu Y., Bai Y. (2019). Metformin Inhibits the NLRP3 Inflammasome via AMPK/mTOR-Dependent Effects in Diabetic Cardiomyopathy. Int. J. Biol. Sci..

[103] Bharath L.P., Agrawal M., McCambridge G., Nicholas D.A., Hasturk H., Liu J., Jiang K., Liu R., Guo Z., Deeney J. (2020). Metformin Enhances Autophagy and Normalizes Mitochondrial Function to Alleviate Aging-Associated Inflammation. Cell Metab..

[104] Bai J., Liu C., Zhu P., Li Y. (2020). Novel Insights Into Molecular Mechanism of Mitochondria in Diabetic Cardiomyopathy. Front. Physiol..

[105] Tahrir F.G., Langford D., Amini S., Mohseni Ahooyi T., Khalili K. (2019). Mitochondrial quality control in cardiac cells: Mechanisms and role in cardiac cell injury and disease. J. Cell. Physiol..

[106] Chen Y., Xin Y., Cheng Y., Liu X. (2022). Mitochondria-Endoplasmic Reticulum Contacts: The Promising Regulators in Diabetic Cardiomyopathy. Oxidative Med. Cell. Longev..

[107] Morales P.E., Arias-Durán C., Ávalos-Guajardo Y., Aedo G., Verdejo H.E., Parra V., Lavandero S. (2020). Emerging role of mitophagy in cardiovascular physiology and pathology. Mol. Asp. Med..

[108] Titus A.S., Sung E.A., Zablocki D., Sadoshima J. (2023). Mitophagy for cardioprotection. Basic. Res. Cardiol..

[109] Zhou R., Zhang Z., Li X., Duan Q., Miao Y., Zhang T., Wang M., Li J., Zhang W., Wang L. (2025). Autophagy in High-Fat Diet and Streptozotocin-Induced Metabolic Cardiomyopathy: Mechanisms and Therapeutic Implications. Int. J. Mol. Sci..

[110] Cai W., Chong K., Huang Y., Huang C., Yin L. (2024). Empagliflozin improves mitochondrial dysfunction in diabetic cardiomyopathy by modulating ketone body metabolism and oxidative stress. Redox Biol..

[111] Tian J., Zhang M., Suo M., Liu D., Wang X., Liu M., Pan J., Jin T., An F. (2021). Dapagliflozin alleviates cardiac fibrosis through suppressing EndMT and fibroblast activation via AMPKα/TGF-β/Smad signalling in type 2 diabetic rats. J. Cell. Mol. Med..

[112] Yang C., Xiao C., Ding Z., Zhai X., Liu J., Yu M. (2024). Canagliflozin Mitigates Diabetic Cardiomyopathy Through Enhanced PINK1-Parkin Mitophagy. Int. J. Mol. Sci..

[113] Zhang Z., Zhang X., Meng L., Gong M., Li J., Shi W., Qiu J., Yang Y., Zhao J., Suo Y. (2021). Pioglitazone Inhibits Diabetes-Induced Atrial Mitochondrial Oxidative Stress and Improves Mitochondrial Biogenesis, Dynamics, and Function Through the PPAR-γ/PGC-1α Signaling Pathway. Front. Pharmacol..

[114] Chen X., Wang T., Gao Y., Wang G.A., Guan J., Dai H. (2025). Liraglutide suppresses ferroptosis by upregulation NRF2 in type 2 diabetic cardiomyopathy. Peptides.

[115] Fang P., Ye Z., Li R., She D., Zong G., Zhang L., Xue Y., Zhang K. (2023). Glucagon-Like Peptide-1 Receptor Agonist Protects Against Diabetic Cardiomyopathy by Modulating microRNA-29b-3p/SLMAP. Drug Des. Dev. Ther..

[116] Cai Y.Y., Zou S.Z., Fan C.X., Wu C.Y., Fang S., Li P., Xue Y.M., Guan M.P. (2018). [Exendin-4 alleviates diabetic cardiomyopathy in mice by regulating Sirt1/PGC1α]. Nan Fang Yi Ke Da Xue Xue Bao.

[117] Jin T., Fu X., Liu M., An F. (2023). Finerenone attenuates myocardial apoptosis, metabolic disturbance and myocardial fibrosis in type 2 diabetes mellitus. Diabetol. Metab. Syndr..

[118] Yaghooti H., Mohyadini M., Bathaie S.Z., Dinarvand N., Mohammadtaghvaei N. (2025). Eplerenone alleviates diabetic cardiomyopathy by modulating ER stress, oxidative stress, and NLRP3 inflammasome activation. J. Diabetes Metab. Disord..

[119] Liu W., Gong W., He M., Liu Y., Yang Y., Wang M., Wu M., Guo S., Yu Y., Wang X. (2018). Spironolactone Protects against Diabetic Cardiomyopathy in Streptozotocin-Induced Diabetic Rats. J. Diabetes Res..

[120] Parichatikanond W., Pandey S., Mangmool S. (2024). Exendin-4 exhibits cardioprotective effects against high glucose-induced mitochondrial abnormalities: Potential role of GLP-1 receptor and mTOR signaling. Biochem. Pharmacol..

[121] Pollard C.M., Suster M.S., Cora N., Carbone A.M., Lymperopoulos A. (2022). GRK5 is an essential co-repressor of the cardiac mineralocorticoid receptor and is selectively induced by finerenone. World J. Cardiol..

[122] Liu Y., An C., Liu P., Yang F., Zhao Q. (2023). Comparative safety of sodium-glucose co-transporter 2 inhibitors in elderly patients with type 2 diabetes mellitus and diabetic kidney disease: A systematic review and meta-analysis. Ren. Fail..

[123] Ravindran S., Munusamy S. (2022). Renoprotective mechanisms of sodium-glucose co-transporter 2 (SGLT2) inhibitors against the progression of diabetic kidney disease. J. Cell. Physiol..

[124] Solomon J., Festa M.C., Chatzizisis Y.S., Samanta R., Suri R.S., Mavrakanas T.A. (2023). Sodium-glucose co-transporter 2 inhibitors in patients with chronic kidney disease. Pharmacol. Ther..

[125] Preda A., Montecucco F., Carbone F., Camici G.G., Lüscher T.F., Kraler S., Liberale L. (2024). SGLT2 inhibitors: From glucose-lowering to cardiovascular benefits. Cardiovasc. Res..

[126] Silva Dos Santos D., Polidoro J.Z., Borges-Júnior F.A., Girardi A.C.C. (2020). Cardioprotection conferred by sodium-glucose cotransporter 2 inhibitors: A renal proximal tubule perspective. Am. J. Physiol. Cell Physiol..

[127] Jaiswal A., Yadav P., Rawat P.S., Kaur M., Babu S.S., Khurana A., Bhatti J.S., Navik U. (2025). Empagliflozin in diabetic cardiomyopathy: Elucidating mechanisms, therapeutic potentials, and future directions. Mol. Biol. Rep..

[128] Li C., Zhang J., Xue M., Li X., Han F., Liu X., Xu L., Lu Y., Cheng Y., Li T. (2019). SGLT2 inhibition with empagliflozin attenuates myocardial oxidative stress and fibrosis in diabetic mice heart. Cardiovasc. Diabetol..

[129] Trang N.N., Chung C.C., Lee T.W., Cheng W.L., Kao Y.H., Huang S.Y., Lee T.I., Chen Y.J. (2021). Empagliflozin and Liraglutide Differentially Modulate Cardiac Metabolism in Diabetic Cardiomyopathy in Rats. Int. J. Mol. Sci..

[130] Chen X., Wang T., Gao Y., Wang G., Zhuang L., Liu X., Gong L., Wang M., Dai H., Guan J. (2025). Dapagliflozin attenuates diabetic cardiomyopathy via NRF2 protein upregulation-driven glutathione synthesis to inhibit myocardial ferroptosis. Life Sci..

[131] Takasu T. (2022). The Role of SGLT2 Inhibitor Ipragliflozin on Cardiac Hypertrophy and microRNA Expression Profiles in a Non-diabetic Rat Model of Cardiomyopathy. Biol. Pharm. Bull..

[132] Zhao Y., Lu Z., Zhang H., Wang L., Sun F., Li Q., Cao T., Wang B., Ma H., You M. (2025). Sodium-glucose exchanger 2 inhibitor canagliflozin promotes mitochondrial metabolism and alleviates salt-induced cardiac hypertrophy via preserving SIRT3 expression. J. Adv. Res..

[133] Hopkins S., Baqai F., Gajagowni S., Hickey G. (2025). Direct Cardiac Mechanisms of the Sodium Glucose Co-Transporter 2 Inhibitor Class. J. Cardiovasc. Pharmacol. Ther..

[134] Brown E., Wilding J.P.H., Alam U., Barber T.M., Karalliedde J., Cuthbertson D.J. (2021). The expanding role of SGLT2 inhibitors beyond glucose-lowering to cardiorenal protection. Ann. Med..

[135] Chambers J.M., Croteau D., Pimentel D.R., Gower A.C., Panagia M., Baka T., Qin F., Luptak I., Colucci W.S. (2025). SGLT2 inhibitor upregulates myocardial genes for oxidative phosphorylation and fatty acid metabolism in Gαq-mice. J. Mol. Cell. Cardiol. Plus.

[136] Dabravolski S.A., Zhuravlev A.D., Kartuesov A.G., Borisov E.E., Sukhorukov V.N., Orekhov A.N. (2022). Mitochondria-Mediated Cardiovascular Benefits of Sodium-Glucose Co-Transporter 2 Inhibitors. Int. J. Mol. Sci..

[137] Zhao Z., Pu Y. (2019). Lixisenatide enhances mitochondrial biogenesis and function through regulating the CREB/PGC-1α pathway. Biochem. Biophys. Res. Commun..

[138] Chen S., Wang Q., Bakker D., Hu X., Zhang L., van der Made I., Tebbens A.M., Kovácsházi C., Giricz Z., Brenner G.B. (2024). Empagliflozin prevents heart failure through inhibition of the NHE1-NO pathway, independent of SGLT2. Basic Res. Cardiol..

[139] Scheen A.J. (2018). Cardiovascular Effects of New Oral Glucose-Lowering Agents: DPP-4 and SGLT-2 Inhibitors. Circ. Res..

[140] Zinman B., Wanner C., Lachin J.M., Fitchett D., Bluhmki E., Hantel S., Mattheus M., Devins T., Johansen O.E., Woerle H.J. (2015). Empagliflozin, Cardiovascular Outcomes, and Mortality in Type 2 Diabetes. N. Engl. J. Med..

[141] Verma S., Mazer C.D., Yan A.T., Mason T., Garg V., Teoh H., Zuo F., Quan A., Farkouh M.E., Fitchett D.H. (2019). Effect of Empagliflozin on Left Ventricular Mass in Patients with Type 2 Diabetes Mellitus and Coronary Artery Disease: The EMPA-HEART CardioLink-6 Randomized Clinical Trial. Circulation.

[142] Bhm M., Anker S., Mahfoud F., Lauder L., Filippatos G., Ferreira J.P., Pocock S.J., Brueckmann M., Saloustros I., Schler E. (2023). Empagliflozin, irrespective of blood pressure, improves outcomes in heart failure with preserved ejection fraction: The EMPEROR-Preserved trial. Eur. Heart J..

[143] Solomon S.D., McMurray J.J.V., Claggett B., de Boer R.A., DeMets D., Hernandez A.F., Inzucchi S.E., Kosiborod M.N., Lam C.S.P., Martinez F. (2022). Dapagliflozin in Heart Failure with Mildly Reduced or Preserved Ejection Fraction. N. Engl. J. Med..

[144] Montero-Pérez-Barquero M., Escobar-Cervantes C., Arévalo-Lorido J.C., Conde-Martel A., Salamanca-Bautista P., Manzano-Espinosa L., Formiga F., Díez-Manglano J., Cepeda J.M., González-Franco A. (2023). Projected effectiveness of dapagliflozin in heart failure with reduced ejection fraction in clinical practice. Future Cardiol..

[145] Zelniker T.A., Wiviott S.D., Raz I., Im K., Goodrich E.L., Furtado R.H.M., Bonaca M.P., Mosenzon O., Kato E.T., Cahn A. (2019). Comparison of the Effects of Glucagon-Like Peptide Receptor Agonists and Sodium-Glucose Cotransporter 2 Inhibitors for Prevention of Major Adverse Cardiovascular and Renal Outcomes in Type 2 Diabetes Mellitus. Circulation.

[146] Boshchenko A.A., Maslov L.N., Mukhomedzyanov A.V., Zhuravleva O.A., Slidnevskaya A.S., Naryzhnaya N.V., Zinovieva A.S., Ilinykh P.A. (2024). Peptides Are Cardioprotective Drugs of the Future: The Receptor and Signaling Mechanisms of the Cardioprotective Effect of Glucagon-like Peptide-1 Receptor Agonists. Int. J. Mol. Sci..

[147] Xie S., Zhang M., Shi W., Xing Y., Huang Y., Fang W.X., Liu S.Q., Chen M.Y., Zhang T., Chen S. (2022). Long-Term Activation of Glucagon-like peptide-1 receptor by Dulaglutide Prevents Diabetic Heart Failure and Metabolic Remodeling in Type 2 Diabetes. J. Am. Heart Assoc..

[148] Pandey S., Mangmool S., Parichatikanond W. (2023). Multifaceted Roles of GLP-1 and Its Analogs: A Review on Molecular Mechanisms with a Cardiotherapeutic Perspective. Pharmaceuticals.

[149] Fu Z., Mui D., Zhu H., Zhang Y. (2020). Exenatide inhibits NF-κB and attenuates ER stress in diabetic cardiomyocyte models. Aging.

[150] Kobara M., Toba H., Nakata T. (2022). A Glucagon-like Peptide 1 Analog Protects Mitochondria and Attenuates Hypoxia-Reoxygenation Injury in Cultured Cardiomyocytes. J. Cardiovasc. Pharmacol..

[151] Naruse G., Kanamori H., Yoshida A., Minatoguchi S., Kawaguchi T., Iwasa M., Yamada Y., Mikami A., Kawasaki M., Nishigaki K. (2019). The intestine responds to heart failure by enhanced mitochondrial fusion through glucagon-like peptide-1 signalling. Cardiovasc. Res..

[152] Xiong L., Hu H., Zhu F., Shi H., Fan X., Pan S., Zhu F., Zhang J., Yu Z., Shi Y. (2024). New insight for SS-31 in treating diabetic cardiomyopathy: Activation of mitoGPX4 and alleviation of mitochondria-dependent ferroptosis. Int. J. Mol. Med..

[153] Cai Y.F., Hu W., Wan Y.G., Tu Y., Liu S.Y., Liu W.J., Pan L.Y., Wu K.J. (2025). [Fucoidan sulfate regulates Hmox1-mediated ferroptosis to ameliorate myocardial injury in diabetic cardiomyopathy]. Zhongguo Zhong Yao Za Zhi.

[154] Chen P., Huang X., Wen W., Cao Y., Li W., Huang G., Huang Y., Hu Y., Ma T. (2025). MiR214-3p Ameliorates Diabetic Cardiomyopathy by Inhibiting Ferroptosis. Cardiovasc. Toxicol..

[155] Wang X., Chen X., Zhou W., Men H., Bao T., Sun Y., Wang Q., Tan Y., Keller B.B., Tong Q. (2022). Ferroptosis is essential for diabetic cardiomyopathy and is prevented by sulforaphane via AMPK/NRF2 pathways. Acta Pharm. Sin. B.

[156] Wu S., Zhu J., Wu G., Hu Z., Ying P., Bao Z., Ding Z., Tan X. (2022). 6-Gingerol Alleviates Ferroptosis and Inflammation of Diabetic Cardiomyopathy via the Nrf2/HO-1 Pathway. Oxidative Med. Cell. Longev..

[157] Xie L., Yu Z.Q., Zhang R., Zhang Z.P., Zhang Y., Jin M.Y., Ju Y., Zhao X.H., Guo J.P. (2024). Phloridzin prevents diabetic cardiomyopathy by reducing inflammation and oxidative stress. Eur. J. Pharmacol..

[158] Wang C., Shen S., Kang J., Sugai-Munson A., Xiao X., Zhang Y., Zhu J., Liu Z., McKay T.B., Akeju O. (2025). METTL3 Is Essential for Exercise Benefits in Diabetic Cardiomyopathy. Circulation.

[159] Zhan J., Jin K., Xie R., Fan J., Tang Y., Chen C., Li H., Wang D.W. (2024). AGO2 Protects Against Diabetic Cardiomyopathy by Activating Mitochondrial Gene Translation. Circulation.

[160] Jin L., Geng L., Ying L., Shu L., Ye K., Yang R., Liu Y., Wang Y., Cai Y., Jiang X. (2022). FGF21-Sirtuin 3 Axis Confers the Protective Effects of Exercise Against Diabetic Cardiomyopathy by Governing Mitochondrial Integrity. Circulation.

[161] Diao J., Wei J., Yan R., Fan G., Lin L., Chen M. (2019). Effects of resveratrol on regulation on UCP2 and cardiac function in diabetic rats. J. Physiol. Biochem..

[162] Saini A.S., Taliyan R., Sharma P.L. (2014). Protective effect and mechanism of Ginkgo biloba extract-EGb 761 on STZ-induced diabetic cardiomyopathy in rats. Pharmacogn. Mag..

[163] Zhang X., Zhang Z., Yang Y., Suo Y., Liu R., Qiu J., Zhao Y., Jiang N., Liu C., Tse G. (2018). Alogliptin prevents diastolic dysfunction and preserves left ventricular mitochondrial function in diabetic rabbits. Cardiovasc. Diabetol..

[164] Dugbartey G.J., Wonje Q.L., Alornyo K.K., Adams I., Diaba D.E. (2022). Alpha-lipoic acid treatment improves adverse cardiac remodelling in the diabetic heart—The role of cardiac hydrogen sulfide-synthesizing enzymes. Biochem. Pharmacol..

[165] Zhang B., Zhang J., Zhang C., Zhang X., Ye J., Kuang S., Sun G., Sun X. (2018). Notoginsenoside R1 Protects Against Diabetic Cardiomyopathy Through Activating Estrogen Receptor α and Its Downstream Signaling. Front. Pharmacol..

[166] Sadraei S., Aarabi A., Rajai Firouzabadi S., Alinejadfard M., Mohammadi I., Jolfayi A.G. (2025). Cardiovascular benefits of semaglutide: A systematic review and meta-analysis of randomized controlled trials. BMC Cardiovasc. Disord..

[167] Lenharo M. (2023). Anti-obesity drug also protects against heart disease-what happens next?. Nature.

[168] Perkovic V., Tuttle K.R., Rossing P., Mahaffey K.W., Mann J.F.E., Bakris G., Baeres F.M.M., Idorn T., Bosch-Traberg H., Lausvig N.L. (2024). Effects of Semaglutide on Chronic Kidney Disease in Patients with Type 2 Diabetes. N. Engl. J. Med..

[169] Kolkhof P., Lawatscheck R., Filippatos G., Bakris G.L. (2022). Nonsteroidal Mineralocorticoid Receptor Antagonism by Finerenone-Translational Aspects and Clinical Perspectives Across Multiple Organ Systems. Int. J. Mol. Sci..

[170] Lin K., Wang A., Zhai C., Zhao Y., Hu H., Huang D., Zhai Q., Yan Y., Ge J. (2025). Semaglutide protects against diabetes-associated cardiac inflammation via Sirt3-dependent RKIP pathway. Br. J. Pharmacol..

[171] Zhang L., Ding W.Y., Wang Z.H., Tang M.X., Wang F., Li Y., Zhong M., Zhang Y., Zhang W. (2016). Early administration of trimetazidine attenuates diabetic cardiomyopathy in rats by alleviating fibrosis, reducing apoptosis and enhancing autophagy. J. Transl. Med..

[172] Wang J., Xue H., He J., Deng L., Tian J., Jiang Y., Feng J. (2024). Therapeutic potential of finerenone for diabetic cardiomyopathy: Focus on the mechanisms. Diabetol. Metab. Syndr..

[173] Jia G., Jia Y., Sowers J.R. (2017). Role of mineralocorticoid receptor activation in cardiac diastolic dysfunction. Biochim. Biophys. Acta Mol. Basis Dis..

[174] Batista J.L., Liu Y., Butler J., Del Prato S., Ezekowitz J.A., Lam C.S.P., Marwick T.H., Rosenstock J., Tang W.H.W., Perfetti R. (2024). Racial Differences in Diabetic Cardiomyopathy: The ARISE-HF Trial. J. Am. Coll. Cardiol..

